# Robustness of MEG source imaging in pediatric patients with surgical cavities: A case series

**DOI:** 10.1016/j.ynirp.2026.100423

**Published:** 2026-10-23

**Authors:** Eleanor Hill, Jeremy T. Moreau, Erica Minato, Marc Lalancette, Elisabeth Simard-Tremblay, Bernard Rosenblatt, Jason Karamchandani, Marie-Christine Guiot, Sylvain Baillet, Roy W.R. Dudley

**Affiliations:** a McGill University, 845 Sherbrooke Street West, Montreal, Quebec, H3A 0G4, Canada; b University of Calgary, 2500 University Drive NW, Calgary, Alberta, T2N 1N4, Canada; c Montreal Neurological Institute and Hospital, 3801 University Street, Montreal, Quebec, H3A 2B4, Canada; d Department of Pediatric Neurology, Montreal Children's Hospital, McGill University Health Centers, 1001 Boulevard Décarie, Montréal, Québec, H4A 3H9, Canada; e Department of Pathology, McGill University Health Centre, 1001 Boulevard Décarie, Montreal, Quebec, H4A 3J1, Canada; f Centre de Recherche Du Centre Hospitalier de L’Université de Montréal, 900 Saint-Denis Street, Montreal, Quebec, H2X 0A9, Canada; g Départements de Neurosciences and Médecine, Université de Montréal, Pavillon Paul-G.-Desmarais, 2960 de La Tour Road, Suite 111, Montreal, Quebec, H3T 1J4, Canada; h Department of Pediatric Surgery, Neurosurgery Division, Montreal Children's Hospital, McGill University Health Centers, 1001 Boulevard Décarie, Montréal, Québec, H4A 3H9, Canada

**Keywords:** Magnetic source imaging, Pediatric drug-resistant epilepsy, Pre-surgical evaluation, CSF-Filled cavities, Head models

## Abstract

**Background:**

Magnetoencephalography (MEG) is used to localize the epileptogenic zone (EZ) in children with drug-resistant epilepsy (DRE) undergoing pre-surgical evaluation. However, its accuracy may be compromised in patients with cerebrospinal fluid (CSF)-filled surgical cavities from prior failed resections. Finite element method (FEM) head models of tissue geometry and biophysics may better account for altered anatomy than the commonly used spherical approximations, but the added value in real clinical cases remains unclear.

**Objective:**

We evaluated the impact of head model choice on MEG source imaging (MSI) in pediatric DRE patients with pre-existing surgical cavities undergoing reoperation.

**Methods:**

We retrospectively analysed interictal and ictal events from four patients (ages 9–18) with prior failed epilepsy surgery who underwent MEG before a second or third resection. Dynamic statistical parametric mapping (dSPM) was applied to all six activity datasets. Equivalent current dipole (ECD) estimation retained dipoles for the four interictal datasets; neither ictal dataset yielded dipoles meeting the goodness-of-fit threshold. Source imaging used conventional overlapping-spheres (OS), boundary element method (BEM), advanced 3-layer FEM (3L-FEM), and 4-layer FEM (4L-FEM, including cavities) head models. EZ estimates were compared in terms of volume, EZ-cavity centroid distances, overlap (DICE coefficients), and agreement with the subsequent resection zone (RZ).

**Results:**

Within each mapping approach, distances from dSPM centroids and dipole locations to the cavity differed across head models by less than 1 cm for all but one activity dataset. Pairwise dSPM source-centroid distances reached 1.84 cm, and pairwise dipole distances reached 4.39 cm in one participant's MEG session. OS and BEM dSPM estimates were typically larger in volume than FEM counterparts, with FEM providing more spatially restricted sources. In the seizure-free cases, the dSPM source imaging from 3 out of 4 epileptiform events overlapped with the subsequent RZ. Computationally, OS models were generated within seconds, BEM models in ∼7 min, whereas 4L-FEM required >20 min per head model.

**Conclusions:**

In this pediatric epilepsy case series with surgical cavities, MSI showed broad agreement across simple spherical and advanced realistic head models. These findings did not establish a consistent localization advantage of one head model over another. However, larger prospective cohorts with independent ground truths are needed to validate and generalize these findings across cavity types and EZ locations.

## Highlights

• Studied the impact of head model selection on magnetic source imaging for pediatric drug-resistant epilepsy patients with CSF-filled surgical cavities
• Compared source localization, derived from four head models of varying complexity, of epileptiform events captured by magnetoencephalography
• Found minimal differences in source location between the four head models supporting the usage of magnetoencephalography in the repeat presurgical evaluation of complex cases

## 1 Introduction

Epilepsy surgery is a well-established treatment for drug-resistant epilepsy (DRE) with potential seizure freedom outcomes when the epileptogenic zone (EZ) is correctly localized, focal, and removed completely (Dwivedi et al., 2017; Rosenow and Lüders, 2001; Lüders et al., 2006). However, forming an EZ hypothesis can be challenging in the absence of a clear structural abnormality. Such a situation can arise with focal cortical dysplasia (FCD) – the most common seizure-producing pathological entity in children with DRE (Kabat and Król, 2012; Sisodiya, 2004; Colombo et al., 2012; Urbach et al., 2022). In such cases, post-surgical seizure freedom occurs in only 40-60% of cases and may require further surgery (Wang et al., 2016; Kwon and Kim, 2019).

In specialized centres, magnetoencephalographic (MEG) imaging (Baillet, 2017) contributes to EZ determination by noninvasively mapping the origin of interictal epileptiform discharges (IEDs) and seizures with millisecond resolution (Knowlton and Shih, 2004; Knowlton, 2006; Madaan et al., 2021; Anderson et al., 2014; Stefan et al., 2011). Magnetic source imaging (MSI) of these epileptiform events has been correlated with ground truths (e.g., iEEG and surgery), with several works finding more favourable surgical outcomes when the MSI-identified area is resected (Murakami et al., 2016; Tamilia et al., 2019; Ntolkeras et al., 2022) and localization accuracies of <2 cm when compared to intracranial and simulation ground truths (Tamilia et al., 2019; Afnan et al., 2026; Sdoukopoulou et al., 2025). For a review on electric and magnetic source imaging accuracy of epileptiform events, please see Ref. (Sharma et al., 2019). Furthermore, MEG has proven to be of significant clinical value in poorly-defined cases (Seo et al., 2011; Kreidenhuber et al., 2018; Rampp et al., 2019; Burgess et al., 2019; Maslarova et al., 2023), including in the re-evaluation of patients with an initial poor surgical outcome (El et al., 2018; Shawarba et al., 2021; Kirchberger et al., 1998; Muthaffar et al., 2017; Mohamed et al., 2007). MEG imaging requires a biophysical model of head tissues – a *head model* (Baillet et al., 2001a) – derived from the individual structural anatomy obtained from magnetic resonance imaging (MRI). MEG's head modelling is considerably less sensitive to tissue resistivity and geometry than electroencephalography (EEG) (Brigo et al., 2011). While many EEG-based studies support using realistically shaped head models (Baillet et al., 2001a; Kastner et al., 2007; Waberski et al., 1998; Vatta et al., 2010; Ramon et al., 2006), and call for consideration of anatomical defects and cerebrospinal fluid (CSF) (Ramon et al., 2006; Piai et al., 2024; Depuydt et al., 2026; van den Broek et al., 1998; Vorwerk et al., 2014), there are MEG works deeming such details unnecessary. Previous research has shown that MEG can be less sensitive to tissue alterations caused by craniotomy or previous surgeries (Baillet, 2017; Huiskamp, 2004; Cuffin, 1993). However, recent work by Piastra et al. (2022) has raised concerns about the influence of CSF-filled cavities on MSI in patients with cerebral infarction. Similar concerns may apply when using MEG to re-evaluate epilepsy patients with CSF-filled surgical cavities. Using simulated data, Piastra et al. demonstrated that such cavities can cause source displacement and alter source strength, with larger cavities having a greater impact, observable up to 20 mm away (Piastra et al., 2022). They proposed using the finite element method (FEM) for head modelling in such cases because of the more accurate biophysical account of altered tissues than the simplified spherical models typically used in MEG (Huang et al., 1999; Sarvas, 1987; Desmedt and Chalklin, 1989; Mosher et al., 1999).

Overlapping spheres (OS) is a technique that locally fits a sphere per MEG sensor to the inner-skull surface to create a head model (Huang et al., 1999; Leahy et al., 1998). This technique offers a more accurate head model compared to early iterations (e.g., single-shell) (Huang et al., 1999), but it is still a simplification of the head compared to those derived with anatomical segmentation: boundary element method (BEM) and FEM. BEM uses tissue boundaries to create individually-defined anatomical layers (e.g., head/scalp, inner skull, and outer skull) to generate the head model, with each layer being assigned its own conductivity value (Kybic et al., 2005; Gramfort et al., 2010). FEM head modelling, however, defines these tissues by volume using many tetrahedra to delineate the space between layers (Piastra et al., 2022; Medani et al., 2023). Despite BEM and FEM being more realistic, BEM/FEM modelling is not the most common approach utilized for MEG, with many opting for computationally simpler models.

Given that the likelihood of achieving seizure freedom decreases with each additional surgery (Yardi et al., 2020; Goodman, 2011), and the risk of neurological deficits increases (Grote et al., 2016), optimizing MEG head modelling techniques becomes crucial. This study aimed to evaluate the impact of head model selection on the MEG localization of the EZ in pediatric patients with DRE who had previously undergone failed surgery. We hypothesized that these CSF-filled surgical cavities would negatively affect the accuracy of MSI, but adequate biophysical modelling of head tissues would overcome these challenges.

## 2 Methods

This study was approved by the Research Ethics Board of the McGill University Health Centre (NEU-14-058). Informed written consent was obtained from participants’ parents/legal guardians for MEG testing and participation in this study. Assent from the pediatric participants was obtained where possible.

### 2.1 Participants

Participants were included from a consecutive series of 99 patients with DRE at the Montreal Children's Hospital who underwent MEG recordings as part of their presurgical evaluation between July 2015 and September 2023. Inclusion criteria were: 1) having undergone at least one resection surgery that failed to control their seizures, resulting in an MRI-visible surgical cavity, prior to the MEG session; 2) exhibiting epileptiform activity during the MEG recording; and 3) having undergone another epilepsy surgery following the MEG recording. These criteria were applied at the participant level. Participant D's later MEG session was also included as a longitudinal comparison, although no subsequent surgery followed that recording. Five participants met these criteria; however, one was excluded due to issues with head digitization during the MEG session, leading to inadequate MRI co-registration.

### 2.2 Anatomical data acquisition and processing

Individual 3D T1-weighted MRIs were used to inform MEG imaging with each participant's cortical anatomy. These MRIs were segmented using *BrainSuite* (Shattuck and Leahy, 2002), with manual adjustments to edge and diffusion parameters to maximize the inclusion of relevant voxels in the cortical segmentation around cavity regions. A second set of T1-weighted MRIs was obtained following the additional surgery and co-registered with the original MRIs using the *SPM12* (Ashburner et al., 2021) plugin in *BrainStorm* (Tadel et al., 2011) to assess overlap between the MEG-imaged activity and the subsequent resection zone (RZ). Three-dimensional regions of interest (ROIs) of the original surgical cavities and the subsequent RZs were manually created using *ITK-SNAP* (Yushkevich et al., 2006, 2016).

### 2.3 Magnetoencephalography data acquisition and processing

Simultaneous resting-state scalp EEG (27-channel scalp EEG using the international 10-20 electrode-placement system) and MEG recordings were acquired with participants in a supine position using a 275-channel CTF MEG system at a sampling rate of 2400 Hz. Additionally, electrooculography (EOG) and electrocardiography (ECG) were acquired to record ocular and cardiac signals to assist artifact detection. During data acquisition, the participants' heads were digitized using a Polhemus Fastrak device through *BrainStorm* (Tadel et al., 2011) for co-registration with the participants’ anatomy.

The EEG and MEG data underwent the following pre-processing steps: 1) notch filtering at 60 Hz to attenuate power-line noise, 2) bandpass filtering between 1 and 70 Hz, and 3) resampling to 300 Hz. EEG recordings were reviewed by board-certified pediatric epileptologists (E.S.T. and B.R.) to identify epileptiform activity. In MEG, artifacts from cardiac activity, ocular movements and blinks, and other sources were minimized using signal-space projections (SSPs) (Tesche et al., 1995; Uusitalo and Ilmoniemi, 1997). Noisy channels and data segments with head movements exceeding 5 mm were rejected.

For each participant, OS, BEM, 3-layer FEM (3L-FEM), and 4-layer FEM (4L-FEM) head models were computed using individual MRI volumes with a regular isotropic cortical grid of 5 mm resolution. These head models were selected to investigate head models of simple (OS), intermediate (BEM and 3L-FEM), and advanced (4L-FEM) complexity. OS head modelling was performed using *BrainStorm'*s default parameters (Tadel et al., 2011). For the BEM and 3L-FEM head modelling, we obtained the head, outer skull, and inner skull surfaces using *BrainStorm's* BEM solution with default parameters (i.e., 1922 vertices per layer, 4 mm skull thickness) (Tadel et al., 2011). After visual inspection of these layers confirmed no intersection, the surfaces were then used to create the BEM head model with *OpenMEEG* software (adaptive integration setting used) (Kybic et al., 2005; Gramfort et al., 2010) and conductivity values of 0.33 S/m for the brain, 0.008 S/m for the skull, and 0.43 S/m for the scalp (Vorwerk et al., 2014). The BEM surfaces were also combined to form a 3L-FEM mesh (brain, skull, and scalp) using the *MergeMesh* option of the *Iso2mesh BrainStorm* plugin with default settings (tetrahedral volume of 0.1 and retaining 100% of elements) (Tadel et al., 2011; Tran et al., 2020). This 3L-FEM mesh was then used to create the 3L-FEM head model with *DUNEuro* as available through *BrainStorm* (default FEM solver and source model settings used) (Medani et al., 2021, 2023; Tadel et al., 2011; Schrader et al., 2021), using the same conductivity values as the BEM head modelling. The 4L-FEM head model was created using the same steps as the 3L-FEM, with the addition of the *ITK-SNAP*-defined cavity (Yushkevich et al., 2016) as the fourth surface with a conductivity value of 1.79 S/m for CSF (Vorwerk et al., 2014).

### 2.4 Magnetic source imaging

For each epileptiform event, MSI was performed using each of the four head model types, and two inverse solutions - dynamic statistical parametric mapping (dSPM)(Dale et al., 2000) and equivalent current dipole (ECD) (Hämäläinen et al., 1993) source estimation. ECD was performed using *BrainStorm's dipole modelling* with diagonal noise covariance, for each activity and head model. From this, single-dipole grid-scan estimates were computed using *BrainStorm's dipole scanning* (Tadel et al., 2011) at the time point of interest with scout limitation deactivated. Unconstrained source orientation was employed for all MSI. Noise covariance was estimated from 2-min empty room recordings acquired immediately before resting-state.

#### 2.4.1 Interictal epileptiform discharges

IEDs were epoched from −200 ms to +500 ms relative to the peak of the discharge, DC-offset correction was applied using the first 100 ms of the epoch, and then averaged. Only IEDs with the same EEG topography and morphology were averaged together. The determination of different IED populations was performed by board-certified pediatric epileptologists (B.R. and E.S.T. - cases were divided, causing only one epileptologist to review each case). For IEDs, the source map corresponding to the time point approximately halfway up the ascending slope of the peak discharge was used to estimate the EZ for both dSPM and ECD solutions. This time point was selected to reduce the contribution of propagated epileptiform activity.

In instances where the resting-state data was in multiple continuous segments (e.g., due to head motion resulting in multiple rest segments), dSPM and ECD was conducted per segment's IED average at the same time point. The resulting dSPM segment sources were weighted-averaged together in source space before undergoing thresholding and further analysis (see Section 2.5.1 dSPM source maps). However, the segment ECD solutions remained unique before goodness-of-fit thresholding and then reduced to a representative location (see Section 2.5.2 ECD source maps).

#### 2.4.2 Seizure events

Seizure events were isolated from −10 s to +5 s around seizure onset. DC-offset correction was applied using first 1 s for seizure epochs (i.e., −10 to −9 s). For seizures, the dSPM EZ was estimated from the map of beta (β, 15-35 Hz) power distribution across the cortex. The source power spectra were obtained using Welch's method (1s sliding window, 50% overlap)(Welch, 1967) over the first 2.5 s of seizures. This ictal period was selected to focus on the origins of seizure onset rather than spread (Andrews et al., 2020), and to avoid movement/muscle associated with the seizure semiology. *BrainStorm's dipole* *scanning* (Tadel et al., 2011) was applied over the same 2.5 s worth of individual waveforms bandpassed to the beta (β, 15-35 Hz) band and using the same settings detailed above (section 2.4 Magnetic source imaging). ECD MSI was attempted for ictal events but was unsuccessful as dipoles did not survive the 70% goodness-of-fit threshold implemented (see 2.5.2 ECD source maps).

### 2.5 Assessment of the MEG source maps

#### 2.5.1 dSPM source maps

The resulting dPSM MEG source maps were thresholded to a prespecified value of 75% (Núñez Ponasso et al., 2025) of their respective maximum values (using *SPM12's Imcalc* (Ashburner et al., 2021)). A range of threshold values has been previously used (Tamilia et al., 2019; Núñez Ponasso et al., 2025; Heers et al., 2016; Pellegrino et al., 2018); we opted for 75% to allow for comparison between high-amplitude source estimates. The resulting thresholded sources were used to determine the EZ estimate from each approach (OS-EZ, BEM-EZ, 3L-EZ, and 4L-EZ). When the 75% threshold produced multiple disconnected clusters, the largest cluster by volume within the presumed EZ region was used for quantitative analysis. The presumed EZ regions were defined using the original MEG presurgical evaluations. The subsequent RZs were known at the time of cluster selection. The volumes of each of these EZ estimates derived from the different head models were calculated. We also calculated the Euclidean distances between the centroids of these EZ estimates and the centroid of the surgical cavity. The difference between volumes and distances was determined to assess the similarity of size and location of the magnetic EZ estimates derived from the four head models per epileptiform event. Additionally, DICE coefficients—a measure of spatial overlap between two 3D objects defined as twice the shared/overlapping volume divided by the sum of the object volumes (Dice, 1945; Sørensen, 1948; Carass et al., 2020)—were computed for all pairwise comparisons between the OS-, BEM-, 3L-, and 4L-EZs (i.e., OS-BEM, OS-3L, OS-4L, BEM-3L, BEM-4L, and 3L-4L) (Rorden, 2015). In the seizure-free cases where the magnetic sources overlap with the subsequent RZ, the percentage of EZ-estimates inside and outside the respective RZs were determined (Fig. 8A).

#### 2.5.2 ECD source maps

For all dipoles, a 70% goodness-of-fit threshold was implemented following recommended guidelines (Bagić et al., 2011). Only the surviving dipoles were subsequently used. In the instances where epileptiform activity datasets resulted in multiple dipoles (see above, Section 2.4.1 Interictal epileptiform discharges), a representative dipole location was calculated from the retained segment-level dipoles as unweighted means of the coordinates. Hereafter, *dipole location* refers to either the single retained dipole or this representative location. Retained dipole counts by activity and head model are provided in Supplemental Table 3.

Subsequent analysis of ECD MSI included the calculation of the Euclidean distance between the dipole locations and the cavity centroid. Akin to the dSPM EZ estimates, the differences between distances was determined to assess the similarity of the location of the ECD sources derived from the four head models per epileptiform event. Unfortunately, ictal analysis using a dipolar inverse solution was not possible due to none surviving the 70% goodness-of-fit thresholding. As also stated in previous works, this is potentially due to the poor signal-to-noise ratio of low voltage fast activity ictal onset (Bagić et al., 2011; Katagiri et al., 2023).

As singular dipole locations were used in this study, volumetric-based analyses (e.g., DICE similarity coefficients, differences in volumetric size, percentage inside/outside subsequent RZs) were not performed for ECD solutions.

## 3 Results

Four participants (ages 9–18 years, 1 male) met the inclusion criteria. Participant demographic information including age, sex, previous surgery locations, previous surgery pathology, epileptic activity, number of epileptiform events analysed, post-MEG surgery locations, post-MEG surgery pathology findings, post-MEG seizure freedom status, and duration of seizure freedom is summarized in Table 1. The details and results for each case are outlined below.

**Table 1:** Participant demographic summary.

Pt.	Age (y)	Sex (M/F)	Previous Surgery	Previous Surgery Pathology	Epileptic Activity	N^o^ of Events Included	Post-MEG Surgery	Post-MEG Surgery Pathology	Post-MEG Seizure Freedom (Y/N)	Time Seizure Free
**A**	18	F	Removal of suspected tumor in L. frontal polar	Most consistent with a venous infarct	F7 sharps	48	L. frontal	FCD 3d	Y	9 Years
**B**	10	F	L. frontal	Gliosis	Seizure	1	L. SMA & mid-cingulate	FCD 2b	Y	7 Years
**C**	9	F	L. ant. & post. temporal	FCD 2a	Seizure	1^a^	L. occipital & parietal	FCD 2a	Y	2 Years
					O2/Pz spike and waves	1206				
**D**	13	M	L. ant. temporal	FCD 1b	P7/T7 spike and wave	145	L. post. temporal	Oligodendrocyte hyperplasia	N	NA
	18	M	L. ant. & post. temporal	Oligodendrocyte hyperplasia	P3 spike and wave	403	NA	NA	NA	NA

a Four seizures were captured during Participant C's MEG recording; however, only the highest-quality seizure was included in the analysis. Note that the IED descriptions in the Epileptic Activity column, such as A/B spike and waves, denote a singular IED population that is equally maximal at A and B, rather than two separate IED types. There are two rows for Participant C due to two types of epileptiform activity (interictal epileptiform discharges and seizures) during their single MEG session. Only information concerning the second type of activity is present on the second row. There are also two rows for Participant D as they had two MEG sessions ∼5 years apart. For this case, each demographic column contains the appropriate information, if available. F/M = female/male, L = left, Ant. = anterior, Post. = posterior, SMA = supplementary motor area, FCD = focal cortical dysplasia, Y/N = yes/no.

### 3.1 Case A

At two years of age, Participant A presented with focal impaired consciousness seizures with motor onset involving the right arm. MRI revealed a left frontal lesion suspected to be a brain tumor, for which she underwent surgical resection at an outside hospital. Pathological analysis showed no evidence of a tumor but signs of a previous, likely perinatal, venous infarct. Despite the surgery and multiple trials of various antiseizure medications (ASMs), her seizures persisted and worsened during adolescence. She was referred to the Montreal Children's Hospital neurosurgery clinic at 17 years old. At that time, her EEG revealed sharp IEDs maximal at F7 (Fig. 1Ai). dSPM MSI with OS head modelling of the simultaneously captured events on MEG (Fig. 1Aii), conducted as part of the presurgical evaluation, localized to the left frontal operculum posterior to the existing surgical cavity (Fig. 1B and C, yellow). The second surgery, which expanded the cavity (Fig. 1E), including resection of the OS-EZ, resulted in long-term seizure freedom (9 years at last follow-up). Histopathology revealed FCD type 3d (i.e., cortical lamination abnormalities adjacent to another lesion acquired during early life, such as trauma, ischemic injury, or encephalitis (Najm et al., 2022)).

**Fig. 1:**
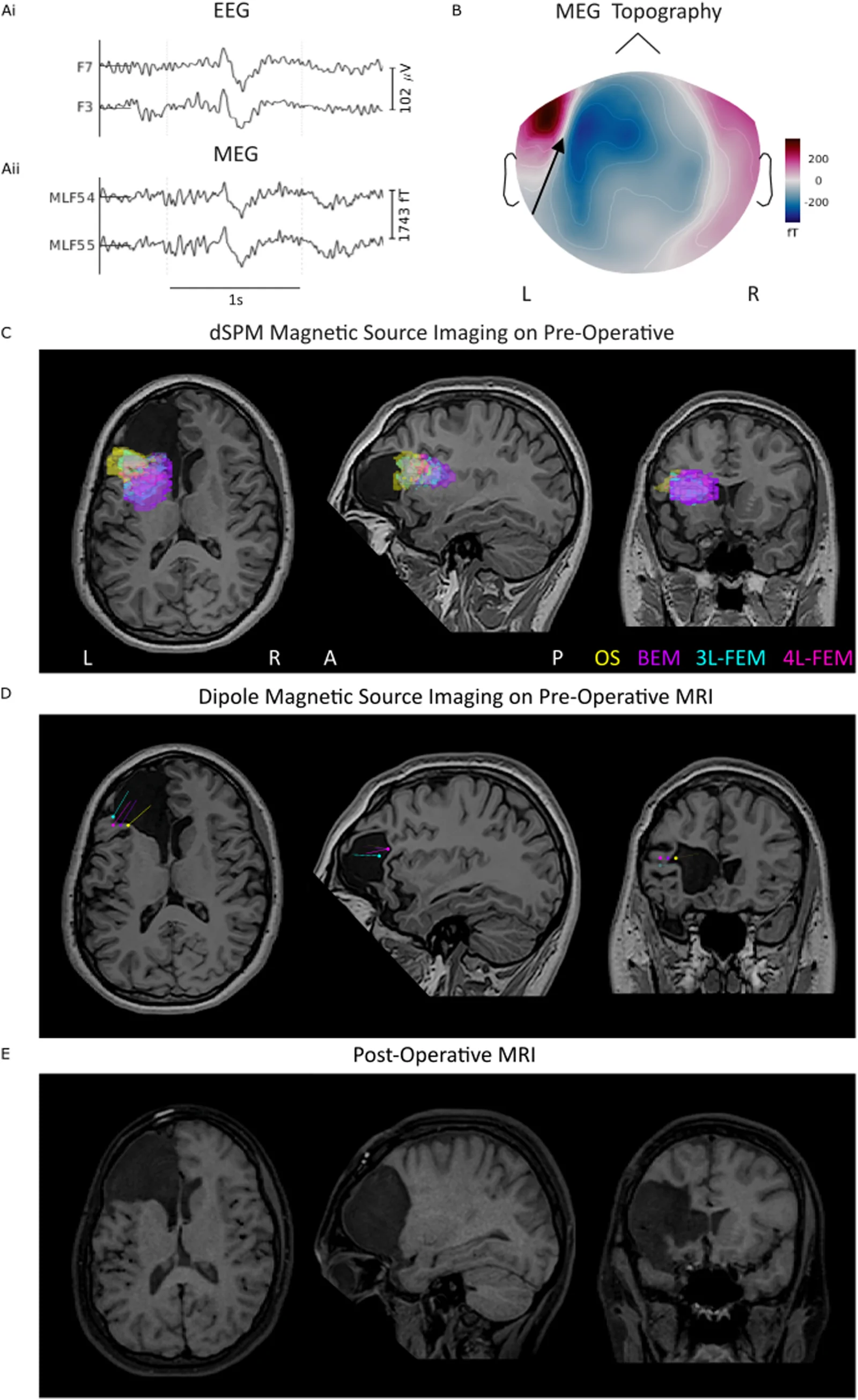
Example traces of Participant A's IED discharges maximal at F7/F3 scalp EEG electrodes captured on Ai) scalp EEG and Aii) MEG. B) MEG topography at ∼1/2 way along the ascending slope of the averaged IEDs. The black arrow indicates the IED localization. Visualization of dSPM (C) and ECD (D) magnetic source imaging derived from OS (yellow), BEM (purple), 3-layer FEM (blue), and 4-layer FEM (magenta) head models overlaid on the participant's pre-operative T1 MRI. E) Post-operative T1 MRI following subsequent surgery.

In this study, we found that dSPM MEG imaging using BEM, 3L-, and 4L-FEM head models also localized to the same region (Fig. 1C), with source-to-cavity centroid distances differing from that of the OS-EZ by less than 1 cm. The dSPM OS-EZ, BEM-EZ, 3L-EZ, and 4L-EZ centroids were found to be 2.18 cm, 2.67 cm, 2.44 cm, and 2.68 cm away from the cavity centroid, respectively. The volumes belonging to OS-EZ and 3L-EZ were similar at 8.49 cm^3^ and 9.89 cm^3^, respectively. However, the 4L-EZ volume was found to be 4.38 cm^3^, only 52% and 44% of OS-EZ and 3L-EZ volumes, respectively. On the other hand, the BEM-EZ volume was much larger at 14.01 cm^3^. The DICE similarity coefficients of these source volumes were ${DICE}_{\;OS-BEM}=0.43$, ${DICE}_{\;OS-3L}=0.62$, ${DICE}_{OS-4L}=0.41$, ${DICE}_{\;BEM-3L}=0.72$, ${DICE}_{\;BEM-4L}=0.46$, and ${DICE}_{3L-4L}=0.58\text{.}$ The ECD MSI derived from all head models also localized to the left frontal operculum posterior to the existing surgical cavity (Fig. 1D). Differences in dipole-to-cavity-centroid distances were also below 1 cm, with the OS-, BEM-, 3L-, and 4L-dipoles were found to be 1.69 cm, 2.04 cm, 2.18 cm, and 2.44 cm from the cavity centroid, respectively.

For case A, all eight EZ estimates lie immediately posterior to the surgical cavity and agree with the RZ that resulted in long-term seizure freedom. Despite the similarity in location, there was some variation in dSPM volume, with the 4L-EZ being the smallest.

### 3.2 Case B

Participant B had suffered from focal motor seizures involving the right arm since the age of 2.5 years and became refractory to ASMs. At five years old, she underwent a subdural grid implantation that failed to localize the EZ but suggested it might partly involve the lateral prefrontal cortex. A small resection in that area did not control her seizures, and pathological analysis found only non-specific gliosis. At ten years old, she was reinvestigated and underwent MEG, during which a seizure was captured in addition to IEDs maximal at C3/Cz and Fp1 on scalp EEG. Only the seizure is analysed in this study due to the small number (<10) (Laohathai et al., 2021; Aydin et al., 2015) of IEDs captured. The seizure involved a fast-activity electrographic onset (Fig. 2Ai & Aii), followed by eye opening 8 s later and clenching of the right fist. Original presurgical OS-MEG mapping of ictal beta band activity localized to the left frontal region posterior and inferior to the existing cavity (Fig. 2B). However, further investigation was required. Subsequent stereo-electroencephalogram (SEEG) implantation captured multiple seizures and confirmed her EZ to be in the deep, posterior aspect of the left supplementary motor area (SMA) and underlying middle cingulate gyrus, just medial to the dSPM OS MSI localization. In the second surgery, these regions were resected (Fig. 2C), which led to long-term seizure freedom (>7 years at last follow-up). FCD type 2b was found on histopathological analysis.

**Fig. 2:**
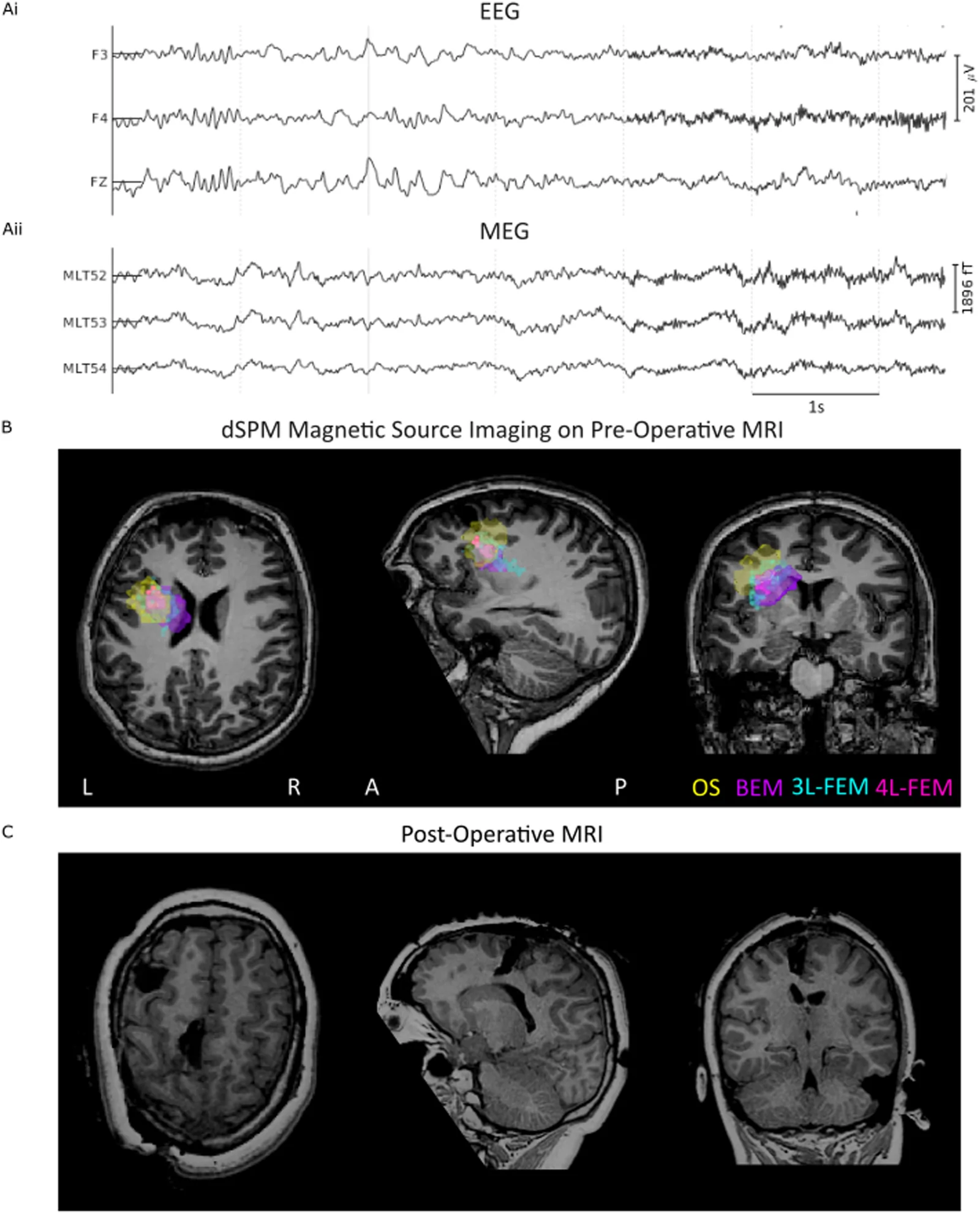
Example traces of Participant B's seizure captured on Ai) scalp EEG and Aii) MEG. B) Visualization of dSPM magnetic source imaging derived from OS (yellow), BEM (purple), 3-layer FEM (blue), and 4-layer FEM (magenta) head models overlaid on the participant's pre-operative T1 MRI. C) Post-operative T1 MRI following subsequent surgery.

In this case, neither the dSPM OS-, BEM-, 3L-, nor 4L-EZ (Fig. 2B) agreed with the subsequent resection. Their source-to-cavity centroid distances were 1.89 cm, 3.23 cm, 2.79 cm, and 2.37 cm, respectively. However, for the dSPM volumes, the OS- and BEM-EZs were notably the largest at 8.61 cm^3^ and 7.63 cm^3^, respectively, while the 3L- and 4L-EZ volumes were found to be 5.25 cm^3^ and 0.87 cm^3^, respectively. With such differences in volume, despite overlapping, DICE values were low at ${DICE}_{\;OS-BEM}=0.08$, ${DICE}_{OS-3L}=0.24$, ${DICE}_{OS-4L}=0.11$, ${DICE}_{\;BEM-3L}=0.36$, ${DICE}_{\;BEM-4L}=0.17$, and ${DICE}_{3L-4L}=0.17$.

While none of the dSPM EZ estimates overlapped with the RZ that resulted in seizure freedom, the original MSI still aided the SEEG implantation planning. Furthermore, there were no large discrepancies between the four dSPM EZ estimates’ locations with respect to the existing surgical cavity. Note, no dipole source localizations are reported for Case B as ictal ECD solutions did not survive the 70% goodness-of-fit threshold applied.

### 3.3 Case C

Participant C began experiencing focal impaired consciousness seizures at ten months of age and was having up to 60-80 seizures per day despite multiple ASM trials. At an outside hospital, she underwent two surgical resections: first of the left anterior temporal lobe, and then, second, an extension to remove the posterior aspect of the temporal lobe. Neither surgery significantly reduced her seizures, and a vagal nerve stimulator was implanted, but seizures persisted. At nine years old, she was referred to the Montreal Children's Hospital for a second opinion due to concerns that her seizures originated from the right hemisphere based on scalp EEG findings of IEDs in the right posterior quadrant. She underwent MEG, during which three epileptiform events were identified, including two IED populations and four similar seizures.

The two IED groups consisted of spike-and-wave discharges maximal at O2/Pz (Fig. 3Ai) and F7/T7/T9. However, for this study, we only analysed the co-localization of her clinically and pathologically proven occipital-parietal EZ (i.e., O2/Pz IEDs). Despite the right O2 EEG finding, MEG concomitant spikes (Fig. 3Aii) localized with dSPM and ECD to the left occipital lobe/midline occipital with all four head models (Fig. 3C and D). For the interictal source volumes, the dSPM OS- and BEM-EZs were once again the largest with volumes of 16.23 cm^3^ and 15.08 cm^3^, respectively. The dSPM 3L- and 4L-EZ volumes were 3.53 cm^3^ and 5.31 cm^3^, respectively. As the four dSPM magnetic sources all overlap with each other, it is not surprising that they share a similar distance from the cavity centroid (5.82 cm, 5.40 cm, 5.67 cm, and 5.47 cm). However, the volumetric differences lent to lower DICE values: ${DICE}_{\;OS-BEM}=0.34$, ${DICE}_{OS-3L}=0.31$, ${DICE}_{OS-4L}=0.36$, ${DICE}_{\;BEM-3L}=0.27$, ${DICE}_{\;BEM-4L}=0.42$, and ${DICE}_{3L-4L}=0.39\text{.}$ The derived dipoles also share very similar locations such that the distances from the cavity centroid (5.98 cm, 5.99 cm, 6.04 cm, and 5.50 cm) differ by less than 0.55 cm.

**Fig. 3:**
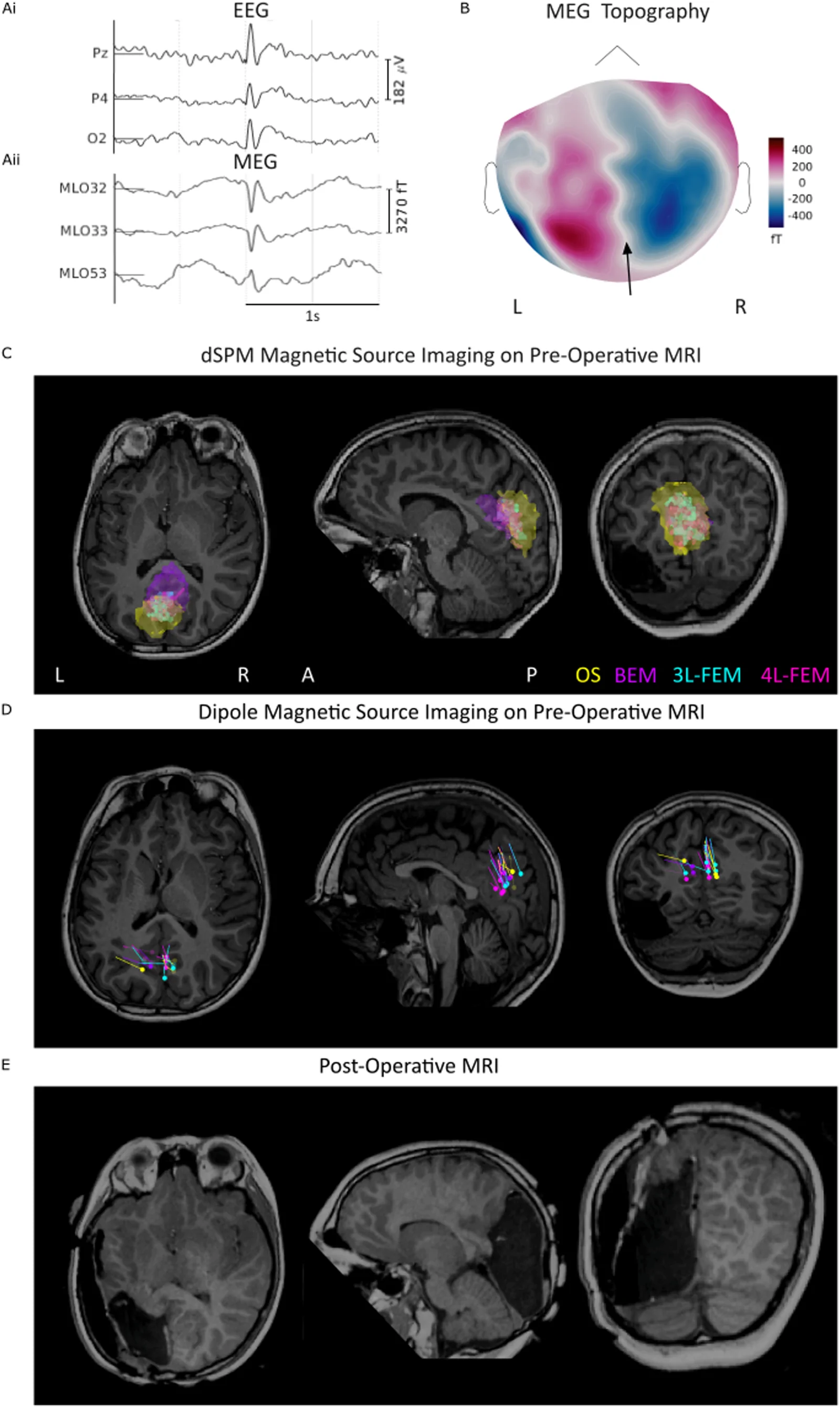
Example traces of Participant C's IED discharges maximal at O2/Pz scalp EEG electrodes captured on Ai) scalp EEG and Aii) MEG. B) MEG topography at ∼1/2 way along the ascending slope of the averaged IEDs. The black arrow indicates the IED localization. Visualization of the dSPM (C) and ECD (D) magnetic source imaging derived from OS (yellow), BEM (purple), 3-layer FEM (blue), and 4-layer FEM (magenta) head models overlaid on the participant's pre-operative T1 MRI. The displayed dipoles represent separate recording-segment estimates; their representative location was used for quantitative analysis as described in Section 2.5.2. E) Post-operative T1 MRI following subsequent surgery.

Seizure onset involved fast activity (Fig. 4Ai & Aii), and MEG mapping of β activity localized the ictal origins to the left temporoparietal-occipital junction with all four head models (Fig. 4B). Four ictal events were recorded during Participant C's MEG session, but only the one of best quality was analysed in this study. The centroids of the OS-, BEM-, 3L-, and 4L-EZs were 4.43 cm, 4.07 cm, 4.01 cm, and 4.06 cm from the cavity centroid, respectively. Similar to the interictal activity, the OS-EZ (5.79 cm^3^) had the largest volume, followed by the BEM-EZ (3.21 cm^3^), 4L-EZ (1.68 cm^3^), and then the 3L-EZ (1.53 cm^3^). The DICE similarity coefficients between the ictal magnetic sources were found to be ${DICE}_{\;OS-BEM}=0.25$, ${DICE}_{OS-3L}=0.29$, ${DICE}_{OS-4L}=0.34$, ${DICE}_{\;BEM-3L}=0.45$, ${DICE}_{\;BEM-4L}=0.30$, and ${DICE}_{3L-4L}=0.42$. Note, no dipole source localizations are reported for Case C's ictal event as ictal ECD solutions did not survive the 70% goodness-of-fit threshold applied.

**Fig. 4:**
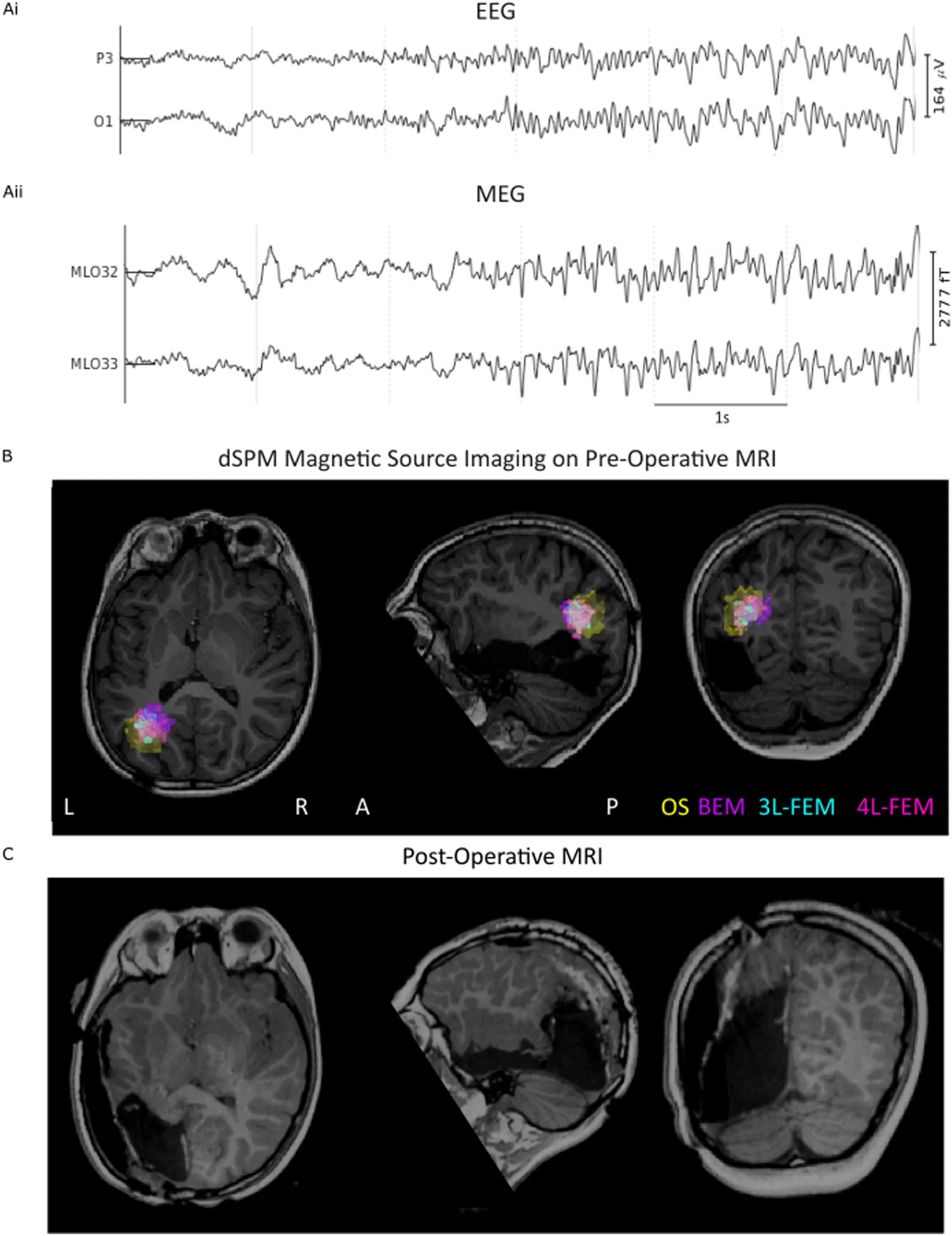
Example traces of Participant C's seizure captured on Ai) scalp EEG and Aii) MEG. B) Visualization of the dSPM magnetic source imaging derived from OS (yellow), BEM (purple), 3-layer FEM (blue), and 4-layer FEM (magenta) head models overlaid on the participant's pre-operative T1 MRI. C) Post-operative T1 MRI following subsequent surgery.

The third surgery (Fig. 3, Fig. 4C), involving a complete occipital-parietal resection (sparing the postcentral gyrus) and extension of the posterior temporal resection, has led to Participant C being seizure-free for more than 2 years without deficits. Histopathological analysis revealed FCD type 2a. The OS-, BEM-, 3L-, and 4L-EZs, from both interictal and ictal activities, all implicated the remaining left occipital lobe (Fig. 3, Fig. 4B) and align with the subsequent RZ.

### 3.4 Case D

#### 3.4.1 First MEG session

Participant D presented at nine years of age with focal impaired consciousness seizures, at times evolving to bilateral tonic-clonic seizures. His 3T MRI revealed subtle signal abnormalities in the left temporal lobe suggestive of FCD, and he underwent a left anterior temporal resection, which had little impact on seizure control, despite revealing a histopathological diagnosis of FCD type 1b. At 13 years of age, he underwent a MEG recording as part of a second presurgical evaluation. The clinical ECD result was part of the original workup but was not included in the present quantitative analysis. The OS MSI, performed with ECD source estimation at the time, localized the P7/T7 spike-and-wave discharges (Fig. 5Ai & Aii) to the left posterior temporal and parietal areas, including the left supramarginal gyrus, and extending toward the occipital lobe (Fig. S1). Participant D's second surgery extended his resection into the posterior inferior temporal area (Fig. 5E). The superior temporal gyrus, inferior parietal lobule, and occipital lobe were spared to avoid language deficits and hemianopsia. Hence, the full extent of the MSI-identified tissue was not removed. Even with this cautious approach, this right-handed patient did have some transient language deficits post-operatively that lasted several weeks. This time, histopathological analysis revealed oligodendrocyte hyperplasia (Lin et al., 2020; Di Giacomo et al., 2021) but no signs of FCD.

**Fig. 5:**
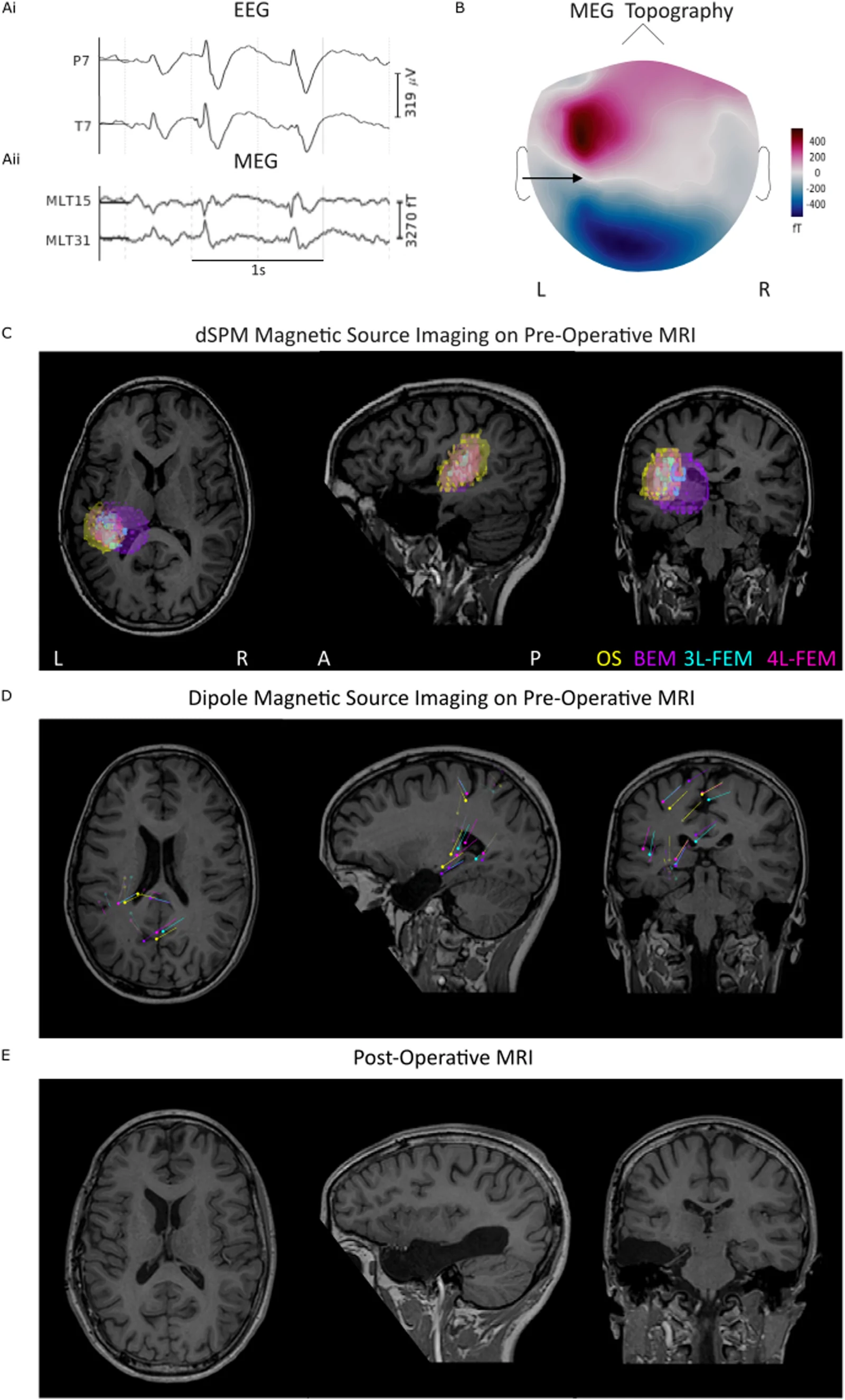
Example traces of Participant D's (first data acquisition) IED discharges maximal at P7/T7 scalp EEG electrodes captured on Ai) scalp EEG and Aii) MEG. B) MEG topography at ∼1/2 way along the ascending slope of the averaged IEDs. The black arrow indicates the IED localization. Visualization of the dSPM (C) and ECD (D) magnetic source imaging derived from OS (yellow), BEM (purple), 3-layer FEM (blue), and 4-layer FEM (magenta) head models overlaid on the participant's pre-operative T1 MRI. The displayed dipoles represent separate recording-segment estimates; their representative location was used for quantitative analysis as described in Section 2.5.2. E) Post-operative T1 MRI following subsequent surgery.

The OS-, BEM-, and FEM-EZs computed retrospectively as part of this study, using dSPM (Fig. 5C), do not notably overlap with the RZ (Fig. 5E) but do with one another. The OS-, BEM-, 3L-, and 4L-EZ centroids were found to be 4.71 cm, 4.48 cm, 4.92 cm, and 4.98 cm away from the cavity centroid, respectively. While similar in location, the four dSPM EZs, once again, had different volumes: 21.63 cm^3^ for the OS-EZ, and almost double for the BEM-EZ at 43.06 cm^3^. While only 4.45 cm^3^ and 5.85 cm^3^ for the 3L- and 4L-EZs, respectively. The DICE similarity coefficients, between the dSPM EZ estimates, were lowest between OS-EZ, BEM-EZ, and the FEM-based EZs and greatest between the 3L- and 4L-EZs: ${DICE}_{\;OS-BEM}=0.49$, ${DICE}_{OS-3L}=0.31$, ${DICE}_{OS-4L}=0.39$, ${DICE}_{\;BEM-3L}=0.17$, ${DICE}_{\;BEM-4L}=0.23$, and ${DICE}_{3L-4L}=0.54$, possibly due to the volume similarity between the 3L- and 4L-EZs. Likewise, the dipoles (Fig. 5D) share similar localizations with one another at 5.88 cm, 5.72 cm, 5.63 cm, and 5.93 cm away from the cavity centroid.

#### 3.4.2 Second MEG session

Participant D's seizures ceased for almost six months after his second surgery, but returned following a missed ASM dose. Despite the resumption of ASMs, seizures persisted. Although these seizures were less intense and less frequent than his original events, he underwent a third presurgical evaluation and a second MEG recording at age 18. This time, the IEDs recorded consisted of spike-and-wave discharges maximal at P3/C3/F3 (Fig. 6Ai & Aii). The dSPM OS-EZ implicated a similar region to that of the first MEG recording – the left posterior superior temporal gyrus and supramarginal gyrus (Fig. 6C). Another surgery has not been performed because it was felt that the risk to language functions was too great.

**Fig. 6:**
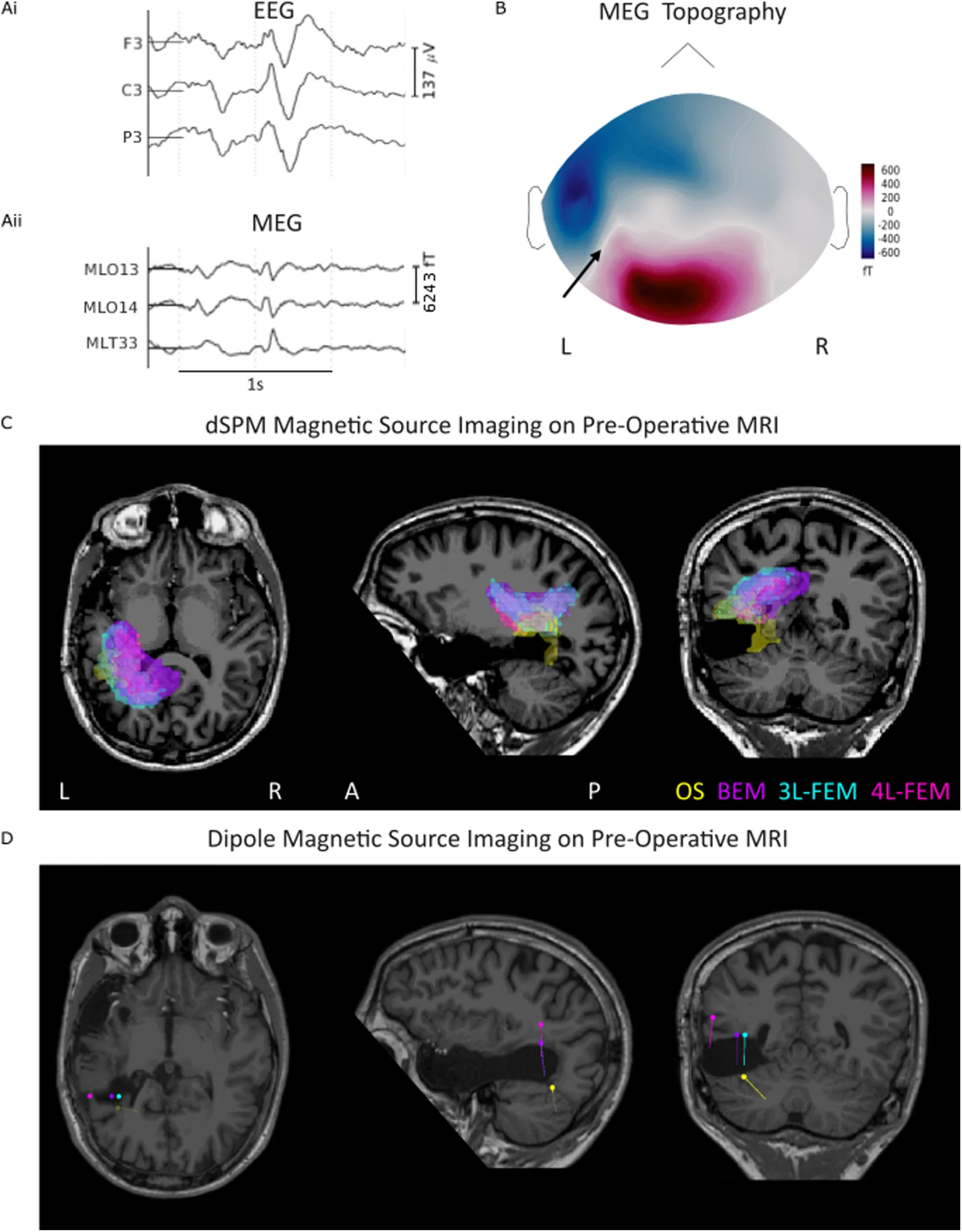
Example traces of Participant D's (second data acquisition) IED discharges maximal at F3/C3/P3 scalp EEG electrodes captured on Ai) scalp EEG and Aii) MEG. B) MEG topography at ∼1/2 way along the ascending slope of the averaged IEDs. The black arrow indicates the IED localization. Visualization of the dSPM (C) and ECD (D) magnetic source imaging derived from OS (yellow), BEM (purple), 3-layer FEM (blue), and 4-layer FEM (magenta) head models overlaid on the participant's pre-operative T1 MRI.

The cavity—source centroid distances had differences of <1 cm, for both ECD and dSPM sources. dSPM OS-, BEM- 3L-, and 4L-EZ centroids were found to be 3.65 cm, 4.59 cm, 4.49 cm, and 3.99 cm away from the cavity centroid, respectively, while dipoles were 4.77 cm, 3.92 cm, 4.00 cm, and 4.59 cm from the cavity centroid. It should be noted that the OS dipole is located on the inferior side of the cavity compared to the superior side like the BEM, 3L, and 4L dipoles (Fig. 6D). However, the cortex identified by OS derived dSPM MSI (Fig. 6C) also extends to the inferior side of the CSF-filled cavity. The dSPM volumes were determined to be 12.00 cm^3^, 29.51 cm^3^, 19.63 cm^3^, and 10.63 cm^3^ for the OS-, BEM-, 3L-, and 4L-EZ, respectively. As such, the greatest DICE similarity coefficient was found between the BEM- and 3L-EZs, and the lowest between the OS- and BEM-EZs: ${DICE}_{\;OS-BEM}=0.25$, ${DICE}_{OS-3L}=0.43$, ${DICE}_{OS-4L}=0.28$, ${DICE}_{\;BEM-3L}=0.54$, ${DICE}_{\;BEM-4L}=0.42$, and ${DICE}_{3L-4L}=0.36$.

While Participant D's surgeries have failed to cure their seizures, and a third has not been performed, the dSPM MSI is relatively stable. Across both MEG acquisitions, dSPM estimates from all four head models indicate involvement of the left posterior superior temporal gyrus and supramarginal gyrus (i.e., Wernicke's language area). ECD estimates differed across head models in the second session, with pairwise distance reaching 4.39 cm (Supplemental Table 2).

Table 2 summarizes the DICE coefficients for each case, and Supplemental Tables 1 and 2 report pairwise dSPM and dipole source-centroid distances, respectively.

**Table 2:** Summary table of the DICE similarity coefficients between dSPM magnetic sources derived from overlapping spheres (OS), boundary element method (BEM), 3-layer finite element method (3L-FEM), and 4-layer FEM (4L-FEM) head models for each participant and their activity. Ci = Participant C's interictal activity, Cii = Participant C's ictal activity, Di = Participant D's activity from their first MEG recording, and Dii = Participant D's activity from the second MEG recording.

***Participant***	DICE Similarity Coefficients					
	***OS:BEM***	***OS:3L-FEM***	***OS:4L-FEM***	***BEM:3L-FEM***	***BEM:4L-FEM***	***3L-FEM:4L-FEM***
**A**	0.43	0.62	0.41	0.72	0.46	0.58
**B**	0.08	0.24	0.11	0.36	0.17	0.17
**Ci**	0.34	0.31	0.36	0.27	0.42	0.39
**Cii**	0.25	0.29	0.34	0.45	0.30	0.42
**Di**	0.49	0.31	0.39	0.17	0.23	0.54
**Dii**	0.25	0.43	0.28	0.54	0.42	0.36

### 3.5 Volumes and distances

The dSPM volumes of the sources derived from the four different head models vary, with the typical trend being that OS- and BEM-EZs are larger than the FEM-derived counterparts, as highlighted in Fig. 7Ai. Out of the six epileptiform events, OS- and BEM-EZs were the largest for 3/6 events each. The smallest EZ volume was equally 3L- and 4L-EZs, also with 3/6 events each. When comparing the volumes (Fig. 7Aii), the largest absolute maximum/minimum differences occurs for BEM-vs 3L-EZs (38.61/1.68 cm^3^) and BEM-vs 4L-EZs (37.21/1.53 cm^3^) while the smallest exists for 3L-vs 4L-EZs (9.00/0.15 cm^3^). When looking at the second-largest volume difference values, we find these are for BEM-vs 4L-EZs (18.88 cm^3^) and OS- vs BEM-EZs (17.51 cm^3^). While the second smallest volume difference value still belongs to 3L-vs 4L-EZs (5.50 cm^3^).

**Fig. 7:**
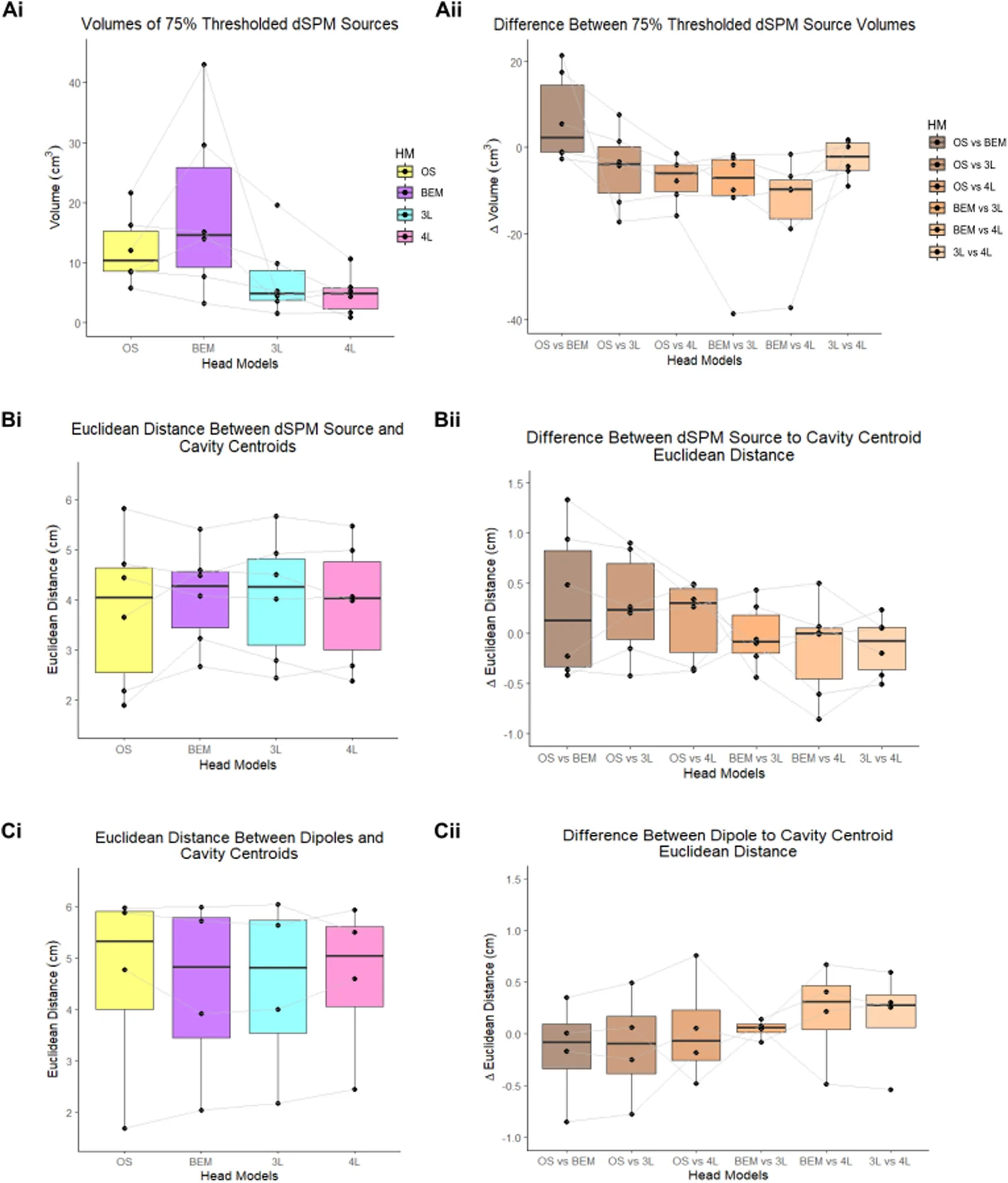
Boxplots of Ai) the volumes (cm^3^) of the 75% thresholded dSPM magnetic sources resulting from OS (yellow), BEM (purple), 3-layer FEM (3L, blue), and 4-layer FEM (4L, magenta) head models; Aii) the differences between dSPM source volumes (cm^3^) derived from OS, BEM, 3-layer FEM, and 4-layer FEM; Bi) the Euclidean distance between the centroids of the dSPM source volumes derived from each head model and the respective cavity centroids (cm); Bii) the difference between dSPM source-cavity centroid distances (cm); Ci) the Euclidean distance between dipole locations derived from OS, BEM, 3-layer FEM, and 4-layer FEM and cavity centroids (cm); Cii) the difference between dipole and cavity centroid distances (cm). Panels A and B include six activity datasets from four participants; panel C includes four interictal datasets from three participants, as neither ictal dataset yielded retained dipoles. Inall plots, the grey lines connect like epileptiform activity and participants across head models. Differences between metrics A vs B are calculated by B−A.

**Fig. 8:**
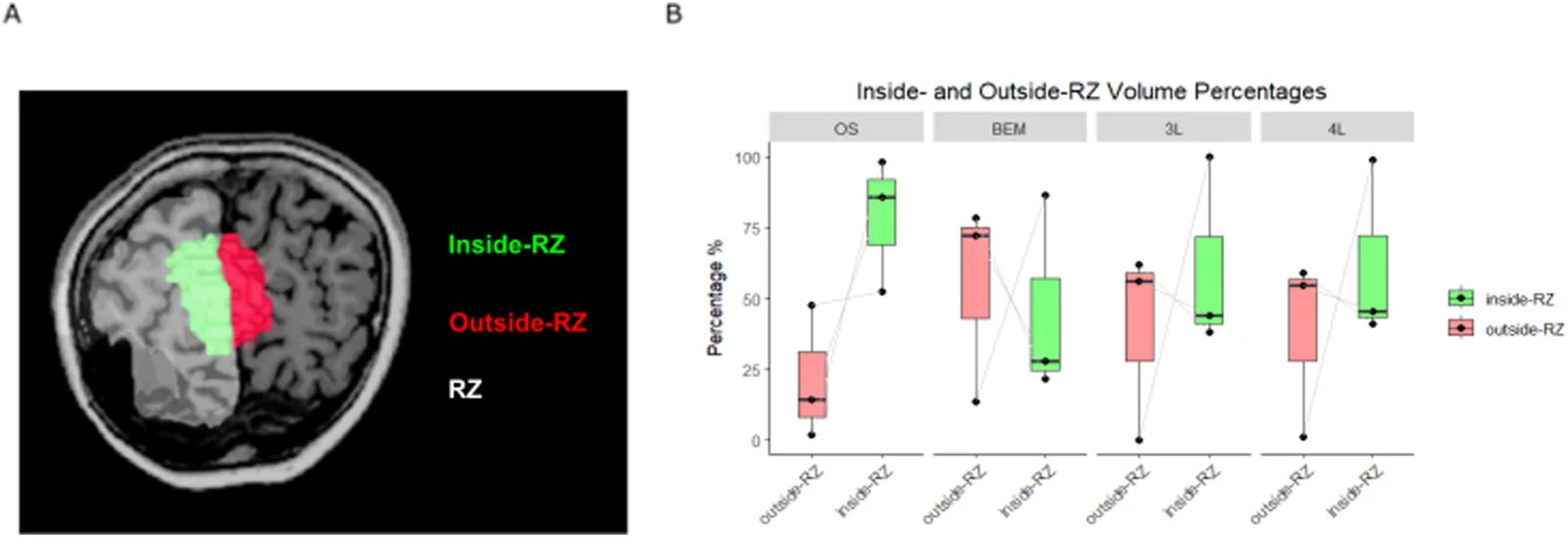
A) schematic of the definition of inside-RZ (EZ-estimate vertices within the subsequent resection zone, RZ) and outside-RZ (EZ-estimate vertices outside the subsequent RZ) in green and red, respectively. The amount of inside- and outside-RZ is presented as percentages of the EZ estimate (i.e., the number of vertices in(out)side of the RZ divided by the total number of EZ-estimate vertices). B) Boxplots of the percentage of the dSPM volumes derived from each head model (for the three activity datasets with dSPM-RZ overlap: A, Ci, and Cii) that are inside-RZ (green) and outside-RZ (red).

Across all epileptiform events and inverse solutions, absolute source–cavity centroid distances ranged from approximately 1.7-6 cm (Fig. 7Bi & Ci), and, for each activity, the distances derived from the four head models differed by less than 1 cm in all but one instance where the distances differed by 1.34 cm (Fig. 7Bii & Cii). These differences in distance to a common reference are distinct from pairwise distances between source estimates, which reached 1.84 cm for dSPM centroids and 4.39 cm for dipole locations (Supplemental Tables 1 and 2).

Given the similarity of localization and size of dSPM 3L- and 4L-EZs, we aimed to disentangle the effects of mesh geometry from cavity conductivity; we therefore constructed a “grey-matter cavity” FEM model that preserved the 4L mesh geometry but set the cavity conductivity equal to grey matter. DICE coefficients between these dSPM GM-cavity sources and the original 3L- and 4L-EZs were <1 in all cases, suggesting that both mesh geometry and CSF conductivity may contribute to the subtle differences observed between FEM models (Fig. S2).

### 3.6 Inside/outside RZ volumes

Three of the participants included in this study are seizure-free, from whom four epileptiform events were recorded. The dSPM MSI of these activities overlapped with the subsequent RZs in 3/4 instances. From these three overlapping instances, the percentage of the EZ-estimate volumes derived from each of the four head models inside and outside the respective RZs can be determined. The inside-RZ percentage is defined as the number of EZ-estimate vertices that overlap with the RZ divided by the total number of EZ-estimate vertices, while the outside-RZ percentage is the number of EZ-estimate vertices outside of the RZ divided by the total EZ-estimate vertices, as demonstrated in Fig. 8A. Out of the four head models, sources derived from the OS head model were found to have a greater inside-RZ percentage (Fig. 8B).

From these three participants, the ECD MSI of two interictal datasets was performed. All of these dipole locations were found to be on the edge of the subsequent RZs.

## 4 Discussion

In this case series of patients who had undergone failed epilepsy surgery prior to their MEG recording, we assessed the impact of fluid-filled surgical cavities on MEG source localization. We evaluated the MEG EZ estimates derived from two different inverse solutions (dSPM and ECD) and four head models (OS, BEM, 3L-FEM, 4L-FEM), ranging in complexity, in terms of location, volume, and RZ agreement. We did so using real data (IEDs and seizures) acquired during patients' repeat presurgical evaluations and open-source tools that were readily available as plug-ins or compatible with the neuroimaging software *BrainStorm* (Medani et al., 2021, 2023; Shattuck and Leahy, 2002; Ashburner et al., 2021; Tadel et al., 2011; Yushkevich et al., 2006, 2016; Tran et al., 2020; Schrader et al., 2021; Kybic et al., 2005; Gramfort et al., 2010). The primary findings from this study are: 1) both the dSPM and ECD source estimates overlapped across head models (except for the OS model showing something slightly different than the other models (bottom vs top of the cavity) for participant D's second MEG session); 2) these comparisons did not establish a consistent localization advantage of cavity-aware over cavity-unaware modelling for presurgical planning; 3) dSPM OS-EZ and BEM-EZ volumes were generally larger than FEM-EZs after thresholding, with FEM providing more spatially restricted estimates.

Across all participants and epileptiform activities, dSPM estimates from the four head models implicated broadly similar cortical regions. ECD estimates were broadly concordant for Participants A and C and Participant D's first session, but were more divergent across head models in Participant D's second session (Supplemental Table 2). Qualitative inspection showed the shared location between the OS-, BEM-, 3L-, and 4L-EZs. Quantitatively, the absolute EZ-cavity centroid distances ranged from approximately 1.7-6 cm across epileptiform events. For each event, the source-cavity centroid distances derived from the four head models differed by less than 1 cm except for one event with a difference of 1.34 cm. All DICE similarity coefficients between dSPM EZ estimates were >0, confirming overlap with one another.

Overall, head models produced broadly similar dSPM estimates in this series, but the dipole discrepancy in Participant D's second session precludes a general conclusion that head model choice has no material effect on localization. The broad dSPM agreement is consistent with prior work suggesting that MEG is relatively insensitive to moderate anatomical and conductivity heterogeneities (Gross, 2019; Corsi and Colliot, 2023; Lopes, 2013) and with an EEG-centred study by Birot et al., who found similar epileptiform EEG source imaging (ESI) localization between spherical (3-shell), BEM (brain, skull, scalp), and FEM (brain, CSF, skull, scalp) head models. However, the authors note that accurate MRI segmentation and labelling of lesions is important for FEM head modelling, but do not explicitly mention surgical cavities (Birot et al., 2014).

Our findings of source concordance across head models align with magnetic head modelling studies outside of this repeat surgery situation. Scheler et al. found, for their epilepsy cohort, no compelling differences in source location derived from 3-layer BEM and 3-spherical shells head models (Scheler et al., 2007). In a phantom study, Leahy et al. typically found spherical- and BEM-based MSI to have similar localization error/performance (Leahy et al., 1998). Haueisen et al. used FEM to investigate the impact of 13 tissue resistivities. Their main MSI findings include that the magnitude of the MEG signal is altered by the resistivity of tissues neighbouring the source (e.g., the grey matter, white matter, and CSF), particularly the grey matter. However, the topography remained relatively unchanged, implicating that such details may not be vital for source localization purposes. Unfortunately, quantification of the source localization was not performed in that study (Haueisen et al., 1997).

Here, we report one case of seizure persistence (Case D). While the first surgery had little impact, the second resulted in temporary seizure relief and long-term overall improvement, suggesting a disruption of the epileptic network. However, neither surgery completely resected the MSI-identified cortex and, to date, a third surgery has not been performed. Hence, we can only speculate that MSI identified a clinically plausible EZ that could not be fully resected because of eloquent-cortex constraints (Sindhu et al., 2023; Vadera et al., 2013). It is worth noting that the MSI-identified cortex has not been removed due to preserving eloquent cortex (visual and language function for Participant D), rather than being discordant with the clinical profile. Across both MEG sessions and four head models, dSPM implicated the left posterior superior temporal and parietal regions, including the left supramarginal gyrus. This pattern persisted across recordings conducted 5 years apart, despite different cavity sizes; however, ECD estimates were more divergent across head models in the second session.

In the seizure-free cases, dSPM MSI of 3 of 4 epileptiform activities agreed with the subsequent RZ. In the singular seizure-free case where the RZ was not identified through MSI (Case B), MEG still assisted SEEG implantation planning (because the dSPM MSI-identified areas were just lateral to the subsequently revealed EZ, leading to implantation of electrodes in and around this area), which resulted in EZ identification and a successful surgery. The lack of overlap between dSPM MSI and the RZ in this case is unlikely to reflect head model selection and instead potentially reflects the limited sensitivity of MEG to deep midline sources (Baillet, 2017; Afnan et al., 2026; Jahromi et al., 2026) like those in the deep posterior SMA and middle cingulate gyrus as seen in this case. In all three seizure-free cases, MSI contributed meaningfully to presurgical planning despite the presence of CSF-filled cavities, further supporting the use of MEG for surgical re-evaluation (El et al., 2018; Shawarba et al., 2021; Kirchberger et al., 1998; Muthaffar et al., 2017; Mohamed et al., 2007), although these retrospective comparisons cannot establish that cavities had no effect on localization accuracy. Instead, our findings align with a case by Huiskamp who found a small localization change (5.5 mm) in MSI when adding CSF to their brain BEM head model (Huiskamp, 2004). However, comparison to this study is limited given the different layers used for BEM head modelling (brain + CSF compared to our brain + skull + scalp), and when CSF was included in our study it was for FEM head modelling only. Nonetheless, our work is in keeping with previous studies demonstrating the usefulness of MEG in repeat presurgical evaluation despite previous surgery (El et al., 2018; Shawarba et al., 2021; Kirchberger et al., 1998; Muthaffar et al., 2017; Mohamed et al., 2007). While these works focused on the clinical yield of MSI rather than the parameters involved, some include head model details that state the use of spherical head models (Kirchberger et al., 1998; Muthaffar et al., 2017; Mohamed et al., 2007) and that the success is potentially due to MEG's lack of signal distortion from the different head tissues and surgical alterations (El et al., 2018; Kirchberger et al., 1998; Mohamed et al., 2007).

Although dSPM source estimates generally overlapped across FEM, OS, and BEM head models, the extent of cortex identified, i.e., the volumes of the dSPM EZ estimates, varied. For all epileptiform events, either the OS- or BEM-EZ volumes were found to be the greatest, while either the 3L- or 4L-EZs were the smallest. In 4/6 epileptiform events, both OS- and BEM-EZ volumes were greater than the FEM-EZ counterparts. Given the general agreement in location but typically smaller volume sizes, dSPM MSI using FEM head modelling may be more conservative while still identifying epileptogenic tissue. This difference may affect implantation planning of intracranial electrodes and resective surgery planning. However, the potential clinical significance requires validation in larger cohorts and across different thresholding methods beyond the prespecified 75% used in this study (e.g., Otsu thresholding (Otsu, 1979), or using the 75th percentile, etc.).

The generally smaller thresholded volumes obtained with FEM should also be considered alongside the computational cost. The generation of the OS head models took <10 s each, BEM head model computation time was ∼7 min, whereas the 3L- and 4L-FEM head models with *DUNEuro* (Medani et al., 2021, 2023; Schrader et al., 2021) took an average of 18.21 min and 23.98 min, respectively, when using a computer with MSI X299 SLI Plus motherboard, Intel Core i9-7920X processor, 128 GB RAM, NVIDIA GeForce GTX 1080 Ti GPU, and Ubuntu 22.04.5 LTS operating system. In the situation where multiple head models are required (e.g., large participant movement resulting in meaningfully different head positions), FEM head model computation could easily become >1 h, whereas OS computation could take <5 min. Despite the difference found here, this would not be considered a substantial practical limitation.

Fundamentally, FEM is a more sophisticated head modelling technique than OS and BEM and typically produced smaller thresholded dSPM volumes in this series. However, these results do not offer a clear advantage for FEM in this study. As such, it is difficult to recommend one head model method over the other. Rather, epileptiform MSI is a matter of striking a balance between model complexity, accuracy, and time constraints. The choice of head modelling technique is for the user to select in their given circumstances, especially for surgical planning.

### 4.1 Limitations

While this small case series includes a unique set of participants of varying surgical histories, cavity sizes, pathologies, and epileptiform activities, including IEDs and seizures, the limited number of participants means we were unable to perform group-level statistical analyses drawing relationships between the head model differences and other features (e.g., sex, head size, cavity size, cavity location, type of subsequent surgery (i.e., extension or secondary location), post-surgical seizure outcome, etc.). As this was a retrospective study, demographics were based on convenience rather than planned recruitment. As a result, there is no confirmed EZ for Participant D, the surgical cavities are not evenly distributed between hemispheres (they are all left-sided, though in different lobes), and the cohort includes only one case in which the follow-up surgery occurred at a different site rather than as an extension of the original cavity.

OS head modelling was used at the time of the original pre-surgical evaluation MSI, whereas the BEM and FEM techniques were retrospectively applied to the epileptiform activity. Therefore, there may be bias towards OS-EZs in the inside/outside-RZ analysis. Furthermore, the CSF-filled cavities and subsequent RZs were manually delineated, which comes with the limitation of human error, particularly when the “post-operative” MRI was performed intra-operatively, where brain shift can be present. Our head modelling pipeline also relies on good-quality individual structural MRIs and co-registration with MEG, and manual segmentations of key tissue boundaries, including surgical cavities, which may not be available or practical in all clinical centres.

Another limitation is the thresholding technique implemented on dSPM. The threshold of 75% of their respective maximum values was prespecified for comparison between higher-amplitude source estimates derived from different head models. However, as briefly stated above, we acknowledge that other thresholding methods are available and should be explored in future works.

The authors recognize that there are more head model options than those addressed in this study (e.g., Huang et al., 1999; Mosher et al., 1999; Lalancette et al., 2011; Berg and Scherg, 1994) and multiple inverse (i.e., source) solutions for MSI (e.g., Dale et al., 2000; Pascual-Marqui, 2002; Baillet et al., 2001b; Jaiswal et al., 2020). While we recognize that this may be a limitation given the unsuccessful ECD MSI of the seizures included in this case series, we did not explore an exhaustive combination of forward and inverse solutions. The aim of this study was to investigate the impact of using a FEM rather than the simple OS and commonly used BEM head models within the MSI practices at our centre in the context of pediatric drug-resistant epilepsy patients with surgical cavities and clinical reference information (i.e., essentially a combination of positive pathology and long-term seizure freedom). Future works, with larger participant populations, could investigate such matters and determine relationships with demographic information such as those listed above. Future work may also broaden the question to other complex anatomies with CSF-filled regions not caused by surgery (e.g., cysts) and determine whether similar localizations between head models hold for deep sources.

Finally, this study only includes MSI. While we do note there is limited literature investigating ESI in the specific context of repeat presurgical evaluation of epilepsy patients with existing CSF-filled cavities, we did not include ESI as a) it was not part of the patients’ original presurgical evaluation and b) ESI is readily known to be sensitive to anatomical alterations (Brigo et al., 2011; Piai et al., 2024; Depuydt et al., 2026; van den Broek et al., 1998; Bénar and Gotman, 2002; Jochmann et al., 2011). That is not to say ESI cannot be used in repeat presurgical evaluations. For example, one case study by Karimi-Rouzbahani et al., used ESI in the planning of a third resection for a pediatric patient suffering from hyperkinetic seizures. The ESI was based on HD-EEG with a realistic head model factoring in CSF and, along with other non-invasive imaging, guided the SEEG implantation and eventual resection that resulted in elimination of the hyperkinetic part of their seizures but not seizure freedom (Karimi-Rouzbahani et al., 2024). Therefore, it would be interesting for future work to determine the utility of ESI for EZ-identification in larger cohorts of repeat surgery patients. Furthermore, within the epilepsy community, there is growing interest in combining ESI and MSI (EMSI) to provide one source estimation(Aydin et al., 2015; Chikara et al., 2023; Duez et al., 2019). With MEG and EEG being considered to provide complementary information, as they are sensitive to different sources (Baillet, 2017; Baillet et al., 1999; Eichardt et al., 2012; Yoshinaga et al., 2002), future work could investigate how many anatomical tissues need to be factored into EMSI head modelling for repeat presurgical evaluations with existing CSF-filled cavities.

## 5 Conclusion

We compared MSI localizations across four head models using two different inverse solutions in four pediatric DRE patients with pre-existing surgical cavities. The dSPM and ECD estimates broadly agreed across head models. This small case series did not establish a consistent localization advantage of cavity-aware FEM over OS or BEM. Additionally, OS-based dSPM EZ-estimates overlapped with the RZ for 2 seizure-free cases. These results do not justify recommending one head model over another for presurgical evaluation in this population, nor do they independently establish localization accuracy. However, future work with larger cohorts of this patient type, with independent ground truths for every case, is needed to determine if this holds across different EZ locations.

## CRediT authorship contribution statement

**Eleanor Hill:** Writing – review & editing, Writing – original draft, Visualization, Methodology, Formal analysis, Data curation, Conceptualization. **Jeremy T. Moreau:** Writing – review & editing, Formal analysis, Data curation. **Erica Minato:** Writing – review & editing, Data curation. **Marc Lalancette:** Writing – review & editing, Data curation. **Elisabeth Simard-Tremblay:** Writing – review & editing, Formal analysis. **Bernard Rosenblatt:** Writing – review & editing, Formal analysis. **Jason Karamchandani:** Writing – review & editing, Formal analysis, Data curation. **Marie-Christine Guiot:** Writing – review & editing, Formal analysis, Data curation. **Sylvain Baillet:** Writing – review & editing, Writing – original draft, Visualization, Supervision, Software, Methodology, Funding acquisition, Conceptualization. **Roy W.R. Dudley:** Writing – review & editing, Writing – original draft, Visualization, Supervision, Methodology, Funding acquisition, Data curation, Conceptualization.

## Ethics declaration

Written informed consent to take part in the study and to publish the article has been obtained from all participants or their legal representatives. The privacy rights of participants have been observed.

This study was performed in compliance with relevant laws, regulatory frameworks and guidelines where the research took place. This study was approved by the Research Ethics Board of the McGill University Health Centre. (Approval No. NEU-14-058).

This research follows the CARE guidelines and the CARE checklist.

## Disclosures

None of the authors has any conflict of interest to disclose.

## Declaration of competing interest

None of the authors have competing interests to disclose.

## Data Availability

The authors do not have permission to share data.

## References

[bib1] Afnan J., Fratello M., Bonini F., Medina Villalon S., Cai Z., Lina J.-M. (2026). Assessing strengths and limitations of magnetoencephalography source imaging with intracerebral EEG. Adv. Sci..

[bib2] Anderson C.T., Carlson C.E., Li Z., Raghavan M. (2014). Magnetoencephalography in the preoperative evaluation for epilepsy surgery. Curr. Neurol. Neurosci. Rep..

[bib3] Andrews J.P., Ammanuel S., Kleen J., Khambhati A.N., Knowlton R., Chang E.F. (2020). Early seizure spread and epilepsy surgery: a systematic review. Epilepsia.

[bib4] Ashburner J., Barnes G., Chen C.-C., Daunizeau J., Flandin G., Friston K. (2021).

[bib5] Aydin Ü., Vorwerk J., Dümpelmann M., Küpper P., Kugel H., Heers M. (2015). Combined EEG/MEG can outperform single modality EEG or MEG source reconstruction in presurgical epilepsy diagnosis. PLoS One.

[bib6] Bagić A., Knowlton R., Rose D., Ebersole J. (2011). American clinical Magnetoencephalography society clinical practice guideline 1: recording and analysis of spontaneous cerebral activity. J. Clin. Neurophysiol..

[bib7] Baillet S. (2017). Magnetoencephalography for brain electrophysiology and imaging. Nat. Neurosci..

[bib8] Baillet S., Garnero L., Marin G., Hugonin J.P. (1999). Combined MEG and EEG source imaging by minimization of mutual information. IEEE (Inst. Electr. Electron. Eng.) Trans. Biomed. Eng..

[bib10] Baillet S., Mosher J., Leahy R. (2001).

[bib9] Baillet S., Riera J.J., Marin G., Mangin J.F., Aubert J., Garnero L. (2001). Evaluation of inverse methods and head models for EEG source localization using a human skull phantom. Phys. Med. Biol..

[bib11] Bénar C.G., Gotman J. (2002). Modeling of post-surgical brain and skull defects in the EEG inverse problem with the boundary element method. Clin. Neurophysiol..

[bib12] Berg P., Scherg M. (1994). A fast method for forward computation of multiple-shell spherical head models. Electroencephalogr. Clin. Neurophysiol..

[bib13] Birot G., Spinelli L., Vulliémoz S., Mégevand P., Brunet D., Seeck M. (2014). Head model and electrical source imaging: a study of 38 epileptic patients. Neuroimage, Clin..

[bib14] Brigo F., Cicero R., Fiaschi A., Bongiovanni L.G. (2011). The breach rhythm. Clin. Neurophysiol..

[bib15] Burgess R.C., Levin K.H., Chauvel P. (2019).

[bib16] Carass A., Roy S., Gherman A., Reinhold J.C., Jesson A., Arbel T. (2020). Evaluating white matter lesion segmentations with refined sørensen-dice analysis. Sci. Rep..

[bib17] Chikara R.K., Jahromi S., Tamilia E., Madsen J.R., Stufflebeam S.M., Pearl P.L. (2023). Electromagnetic source imaging predicts surgical outcome in children with focal cortical dysplasia. Clin. Neurophysiol..

[bib18] Colombo N., Tassi L., Deleo F., Citterio A., Bramerio M., Mai R. (2012). Focal cortical dysplasia type IIa and IIb: MRI aspects in 118 cases proven by histopathology. Neuroradiology.

[bib19] Corsi M.C., Colliot O. (2023). Machine Learning for Brain Disorders.

[bib20] Cuffin B.N. (1993). Effects of local variations in skull and scalp thickness on EEG's and MEG's. IEEE (Inst. Electr. Electron. Eng.) Trans. Biomed. Eng..

[bib21] Dale A.M., Liu A.K., Fischl B.R., Buckner R.L., Belliveau J.W., Lewine J.D. (2000). Dynamic statistical parametric mapping: combining fMRI and MEG for high-resolution imaging of cortical activity. Neuron.

[bib22] Depuydt E., Oostenveld R., De Letter M., van Mierlo P., Piai V. (2026). The impact of brain tumors and craniotomy lesions on scalp EEG. Brain Topogr..

[bib23] Desmedt J.E., Chalklin V. (1989). New method for titrating differences in scalp topographic patterns in brain evoked potential mapping. Electroencephalogr. Clin. Neurophysiology Evoked Potentials Sect..

[bib24] Di Giacomo R., Deleo F., Garbelli R., Marucci G., Del Sole A., Dominese A. (2021). Mild malformation of cortical development with oligodendroglial hyperplasia (MOGHE): neurophysiological fingerprints of a new pathological entity. Clin. Neurophysiol..

[bib25] Dice L.R. (1945). Measures of the amount of ecologic association between species. Ecology.

[bib26] Duez L., Tankisi H., Hansen P.O., Sidenius P., Sabers A., Pinborg L.H. (2019). Electromagnetic source imaging in presurgical workup of patients with epilepsy. Neurology.

[bib27] Dwivedi R., Ramanujam B., Chandra P.S., Sapra S., Gulati S., Kalaivani M. (2017). Surgery for drug-resistant epilepsy in children. N. Engl. J. Med..

[bib28] Eichardt R., Güllmar D., Funke M., Haueisen J. (2012). Tangential and radial epileptic spike activity: different sensitivity in EEG and MEG. J. Clin. Neurophysiol..

[bib29] El Tahry R., Wang Z.I., Thandar A., Podkorytova I., Krishnan B., Tousseyn S. (2018). Magnetoencephalography and ictal SPECT in patients with failed epilepsy surgery. Clin. Neurophysiol..

[bib30] Goodman R.R. (2011). AES 2009 annual course: reoperation for medically refractory epilepsy. Epilepsy Behav..

[bib31] Gramfort A., Papadopoulo T., Olivi E., Clerc M. (2010). OpenMEEG: opensource software for quasistatic bioelectromagnetics. BioMedical Engineering OnLine.

[bib32] Gross J. (2019). Magnetoencephalography in cognitive neuroscience: a primer. Neuron.

[bib33] Grote A., Witt J.-A., Surges R., von Lehe M., Pieper M., Elger C.E. (2016). A second Chance—reoperation in patients with failed surgery for intractable epilepsy: long-term outcome, neuropsychology and complications. J. Neurol. Neurosurg. Psychiatr..

[bib34] Hämäläinen M., Hari R., Ilmoniemi R.J., Knuutila J., Lounasmaa O.V. (1993). Magnetoencephalography---theory, instrumentation, and applications to noninvasive studies of the working human brain. Rev. Mod. Phys..

[bib35] Haueisen J., Ramon C., Eiselt M., Brauer H., Nowak H. (1997). Influence of tissue resistivities on neuromagnetic fields and electric potentials studied with a finite element model of the head. IEEE (Inst. Electr. Electron. Eng.) Trans. Biomed. Eng..

[bib36] Heers M., Chowdhury R.A., Hedrich T., Dubeau F., Hall J.A., Lina J.-M. (2016). Localization accuracy of distributed inverse solutions for electric and magnetic source imaging of interictal epileptic discharges in patients with focal epilepsy. Brain Topogr..

[bib37] Huang M.X., Mosher J.C., Leahy R.M. (1999). A sensor-weighted overlapping-sphere head model and exhaustive head model comparison for MEG. Phys. Med. Biol..

[bib38] Huiskamp G. (2004). The 26th Annual International Conference of the IEEE Engineering in Medicine and Biology Society.

[bib39] Jahromi S., Sdoukopoulou G., Chikara R.K., Stufflebeam S.M., Ottensmeyer M.P., De Novi G. (2026). 3D printed pediatric head phantom for assessing deep epileptic sources localization. Comput. Biol. Med..

[bib40] Jaiswal A., Nenonen J., Stenroos M., Gramfort A., Dalal S.S., Westner B.U. (2020). Comparison of beamformer implementations for MEG source localization. Neuroimage.

[bib41] Jochmann T., Güllmar D., Haueisen J., Reichenbach J.R. (2011). Influence of tissue conductivity changes on the EEG signal in the human brain – a simulation study. Z. Med. Phys..

[bib42] Kabat J., Król P. (2012). Focal cortical dysplasia - review. Pol. J. Radiol..

[bib43] Karimi-Rouzbahani H., Vogrin S., Cao M., Plummer C., McGonigal A. (2024). Multimodal and quantitative analysis of the epileptogenic zone network in the pre-surgical evaluation of drug-resistant focal epilepsy. Neurophysiol. Clin..

[bib44] Kastner J., Wagner M., Fuchs M. (2007). Development of volume conductor and source models to localize epileptic foci. J. Clin. Neurophysiol..

[bib45] Katagiri M., Wang Z.I., Hirfanoglu T., Aldosari M.M., Aung T., Wang S. (2023). Clinical significance of ictal magnetoencephalography in patients undergoing epilepsy surgery. Clin. Neurophysiol..

[bib46] Kirchberger K., Hummel C., Stefan H. (1998). Postoperative multichannel magnetoencephalography in patients with recurrent seizures after epilepsy surgery. Acta Neurol. Scand..

[bib47] Knowlton R.C. (2006). The role of FDG-PET, ictal SPECT, and MEG in the epilepsy surgery evaluation. Epilepsy Behav..

[bib48] Knowlton R.C., Shih J. (2004). Magnetoencephalography in epilepsy. Epilepsia.

[bib49] Kreidenhuber R., Demarchi G., Kalss G., Leitinger M., Winkler P.A., Trinka E. (2018). Benefit of magnetic source localization in challenging refractory epilepsies. Z. für Epileptol..

[bib50] Kwon H.E., Kim H.D. (2019). Recent aspects of pediatric epilepsy surgery. J Epilepsy Res.

[bib51] Kybic J., Clerc M., Abboud T., Faugeras O., Keriven R., Papadopoulo T. (2005). A common formalism for the integral formulations of the forward EEG problem. IEEE Trans. Med. Imag..

[bib52] Lalancette M., Quraan M., Cheyne D. (2011). Evaluation of multiple-sphere head models for MEG source localization. Phys. Med. Biol..

[bib53] Laohathai C., Ebersole J.S., Mosher J.C., Bagić A.I., Sumida A., Von Allmen G. (2021). Practical fundamentals of clinical MEG interpretation in epilepsy. Front. Neurol..

[bib54] Leahy R.M., Mosher J.C., Spencer M.E., Huang M.X., Lewine J.D. (1998). A study of dipole localization accuracy for MEG and EEG using a human skull phantom. Electroencephalogr. Clin. Neurophysiol..

[bib55] Lin Y., Xu X., Aung T., Murakami H., Blümcke I., Bingaman W. (2020). Basal temporo-occipital mild malformation of cortical development with oligodendroglial hyperplasia: a multimodal investigation turning non-lesional to lesional epilepsy. Clin. Neurophysiol..

[bib56] Lopes da Silva F. (2013). EEG and MEG: relevance to neuroscience. Neuron.

[bib57] Lüders H.O., Najm I., Nair D., Widdess-Walsh P., Bingman W. (2006). The epileptogenic zone: general principles. Epileptic Disord..

[bib58] Madaan P., Gupta A., Gulati S. (2021). Pediatric epilepsy surgery: indications and evaluation. Indian J. Pediatr..

[bib59] Maslarova A., Zhao Y., Rösch J., Dörfler A., Coras R., Blümcke I. (2023). Surgical planning, histopathology findings and postoperative outcome in MR-negative extra-temporal epilepsy using intracranial EEG, functional imaging, magnetoencephalography, neuronavigation and intraoperative MRI. Clin. Neurol. Neurosurg..

[bib61] Medani T., Garcia-Prieto J., Tadel F., Antonakakis M., Erdbrügger T., Höltershinken M. (2023). Brainstorm-DUNEuro: an integrated and user-friendly finite element method for modeling electromagnetic brain activity. Neuroimage.

[bib60] Medani T., Garcia-Prieto J., Tadel F., Schrader S., Antonakakis M., Joshi A. (2021).

[bib62] Mohamed I.S., Otsubo H., Ochi A., Elliott I., Donner E., Chuang S. (2007). Utility of magnetoencephalography in the evaluation of recurrent seizures after epilepsy surgery. Epilepsia.

[bib63] Mosher J.C., Leahy R.M., Lewis P.S. (1999). EEG and MEG: forward solutions for inverse methods. IEEE (Inst. Electr. Electron. Eng.) Trans. Biomed. Eng..

[bib64] Murakami H., Wang Z.I., Marashly A., Krishnan B., Prayson R.A., Kakisaka Y. (2016). Correlating magnetoencephalography to stereo-electroencephalography in patients undergoing epilepsy surgery. Brain.

[bib65] Muthaffar O., Puka K., Rubinger L., Go C., Snead O.C., Rutka J.T. (2017). Reoperation after failed resective epilepsy surgery in children. J. Neurosurg.: Pediatrics PED.

[bib66] Najm I., Lal D., Alonso Vanegas M., Cendes F., Lopes-Cendes I., Palmini A. (2022). The ILAE consensus classification of focal cortical dysplasia: an update proposed by an ad hoc task force of the ILAE diagnostic methods commission. Epilepsia.

[bib67] Ntolkeras G., Tamilia E., AlHilani M., Bolton J., Ellen Grant P., Prabhu S.P. (2022). Presurgical accuracy of dipole clustering in MRI-negative pediatric patients with epilepsy: validation against intracranial EEG and resection. Clin. Neurophysiol..

[bib68] Núñez Ponasso G., Drumm D.A., Wang A., Noetscher G.M., Hämäläinen M., Knösche T.R. (2025). High-definition MEG source estimation using the reciprocal boundary element fast multipole method. Neuroimage.

[bib69] Otsu N. (1979). A threshold selection method from gray-level histograms. IEEE Trans. Syst. Man Cybern..

[bib70] Pascual-Marqui R.D. (2002). Standardized low-resolution brain electromagnetic tomography (sLORETA): technical details. Methods Find Exp Clin Pharmacol.

[bib71] Pellegrino G., Hedrich T., Chowdhury R.A., Hall J.A., Dubeau F., Lina J.-M. (2018). Clinical yield of magnetoencephalography distributed source imaging in epilepsy: a comparison with equivalent current dipole method. Hum. Brain Mapp..

[bib72] Piai V., Oostenveld R., Schoffelen J.M., Piastra M.C. (2024). The impact of CSF-filled cavities on scalp EEG and its implications. Psychophysiology.

[bib73] Piastra M.C., Oostenveld R., Schoffelen J.M., Piai V. (2022). Estimating the influence of stroke lesions on MEG source reconstruction. Neuroimage.

[bib74] Ramon C., Schimpf P.H., Haueisen J. (2006). Influence of head models on EEG simulations and inverse source localizations. BioMedical Engineering OnLine.

[bib75] Rampp S., Stefan H., Wu X., Kaltenhäuser M., Maess B., Schmitt F.C. (2019). Magnetoencephalography for epileptic focus localization in a series of 1000 cases. Brain.

[bib76] Rorden C. (2015). Nii_dice GitHub. https://github.com/rordenlab/spmScripts/blob/master/nii_dice.m.

[bib77] Rosenow F., Lüders H. (2001). Presurgical evaluation of epilepsy. Brain.

[bib78] Sarvas J. (1987). Basic mathematical and electromagnetic concepts of the biomagnetic inverse problem. Phys. Med. Biol..

[bib79] Scheler G., Fischer M.J.M., Genow A., Hummel C., Rampp S., Paulini A. (2007). Spatial relationship of source localizations in patients with focal epilepsy: comparison of MEG and EEG with a three spherical shells and a boundary element volume conductor model. Hum. Brain Mapp..

[bib80] Schrader S., Westhoff A., Piastra M.C., Miinalainen T., Pursiainen S., Vorwerk J. (2021). DUNEuro—A software toolbox for forward modeling in bioelectromagnetism. PLoS One.

[bib81] Sdoukopoulou G., Jahromi S., Corona L., Papadelis C. (2025). 2025 47th Annual International Conference of the IEEE Engineering in Medicine and Biology Society (EMBC).

[bib82] Seo J.H., Holland K., Rose D., Rozhkov L., Fujiwara H., Byars A. (2011). Multimodality imaging in the surgical treatment of children with nonlesional epilepsy. Neurology.

[bib83] Sharma P., Seeck M., Beniczky S. (2019). Accuracy of interictal and ictal electric and magnetic source imaging: a systematic review and meta-analysis. Front. Neurol..

[bib84] Shattuck D.W., Leahy R.M. (2002). BrainSuite: an automated cortical surface identification tool. Med. Image Anal..

[bib85] Shawarba J., Kaspar B., Rampp S., Winter F., Coras R., Blumcke I. (2021). Advantages of magnetoencephalography, neuronavigation and intraoperative MRI in epilepsy surgery re-operations. Neurol. Res..

[bib86] Sindhu D.M., Mundlamuri R.C., Goutham B., Narayanan M., Raghavendra K., Asranna A. (2023). Role of magnetoencephalography in predicting the epileptogenic zone and post-operative seizure outcome - a retrospective study. Seizure: European Journal of Epilepsy.

[bib87] Sisodiya S.M. (2004). Surgery for focal cortical dysplasia. Brain.

[bib88] Sørensen T.J. (1948).

[bib89] Stefan H., Rampp S., Knowlton R.C. (2011). Magnetoencephalography adds to the surgical evaluation process. Epilepsy Behav..

[bib90] Tadel F., Baillet S., Mosher J.C., Pantazis D., Leahy R.M. (2011). Brainstorm: a user-friendly application for MEG/EEG analysis. Comput. Intell. Neurosci..

[bib91] Tamilia E., AlHilani M., Tanaka N., Tsuboyama M., Peters J.M., Grant P.E. (2019). Assessing the localization accuracy and clinical utility of electric and magnetic source imaging in children with epilepsy. Clin. Neurophysiol..

[bib92] Tesche C.D., Uusitalo M.A., Ilmoniemi R.J., Huotilainen M., Kajola M., Salonen O. (1995). Signal-space projections of MEG data characterize both distributed and well-localized neuronal sources. Electroencephalogr. Clin. Neurophysiol..

[bib93] Tran A.P., Yan S., Fang Q. (2020). Improving model-based functional near-infrared spectroscopy analysis using mesh-based anatomical and light-transport models. Neurophotonics.

[bib94] Urbach H., Kellner E., Kremers N., Blümcke I., Demerath T. (2022). MRI of focal cortical dysplasia. Neuroradiology.

[bib95] Uusitalo M.A., Ilmoniemi R.J. (1997). Signal-space projection method for separating MEG or EEG into components. Med. Biol. Eng. Comput..

[bib96] Vadera S., Jehi L., Burgess R.C., Shea K., Alexopoulos A.V., Mosher J. (2013). Correlation between magnetoencephalography-based “clusterectomy” and postoperative seizure freedom. Neurosurgical Focus FOC.

[bib97] van den Broek S.P., Reinders F., Donderwinkel M., Peters M.J. (1998). Volume conduction effects in EEG and MEG. Electroencephalogr. Clin. Neurophysiol..

[bib98] Vatta F., Meneghini F., Esposito F., Mininel S., Di Salle F. (2010). Realistic and spherical head modeling for EEG forward problem solution: a comparative cortex-based analysis. Comput. Intell. Neurosci..

[bib99] Vorwerk J., Cho J.-H., Rampp S., Hamer H., Knösche T.R., Wolters C.H. (2014). A guideline for head volume conductor modeling in EEG and MEG. Neuroimage.

[bib100] Waberski T.D., Buchner H., Lehnertz K., Hufnagel A., Fuchs M., Beckmann R. (1998). Properties of advanced headmodelling and source reconstruction for the localization of epileptiform activity. Brain Topogr..

[bib101] Wang X., Zhang C., Wang Y., Hu W., Shao X., Zhang J-g (2016). Prognostic factors for seizure outcome in patients with MRI-negative temporal lobe epilepsy: a meta-analysis and systematic review. Seizure.

[bib102] Welch P. (1967). The use of fast Fourier transform for the estimation of power spectra: a method based on time averaging over short, modified periodograms. IEEE Trans. Audio Electroacoust..

[bib103] Yardi R., Morita-Sherman M.E., Fitzgerald Z., Punia V., Bena J., Morrison S. (2020). Long-term outcomes of reoperations in epilepsy surgery. Epilepsia.

[bib104] Yoshinaga H., Nakahori T., Ohtsuka Y., Oka E., Kitamura Y., Kiriyama H. (2002). Benefit of simultaneous recording of EEG and MEG in dipole localization. Epilepsia.

[bib106] Yushkevich P.A., Gao Y., Gerig G. (2016). 2016 38th Annual International Conference of the IEEE Engineering in Medicine and Biology Society (EMBC).

[bib105] Yushkevich P.A., Piven J., Hazlett H.C., Smith R.G., Ho S., Gee J.C. (2006). User-guided 3D active contour segmentation of anatomical structures: significantly improved efficiency and reliability. Neuroimage.

