# Supplementary material for: Robustness of MEG source imaging in pediatric patients with surgical cavities: A case series
Source: Neuroimage Rep. 2026 Oct 23;6(4):100423. doi: 10.1016/j.ynirp.2026.100423 (PMC13715417; doi:10.1016/j.ynirp.2026.100423)
Supplement: Supplementary file 1 [file mmc1.docx]

**Supplemental Material**

**Supplemental Figure 1:** Original equivalent current dipole (ECD) magnetic source imaging performed with an overlapping spheres head model for individual P7/T7 spike and waves captured during Participant D’s first MEG recording. ECDs were computed using the peak of the spikes, and a 70% goodness-of-fit threshold was applied. The color represents goodness-of-fit, whereby the red tones are towards 70% while yellow is towards 100%. These historical clinical dipoles were not included in the present quantitative comparisons. The ECD results displayed here generally agree with the dynamic statistical parametric mapping (dSPM) source localizations shown in the main text (Fig. 5C). Both solutions localized posterior to the surgical cavity, near the left posterior superior temporal and supramarginal gyri; the ECD solution extended farther into the residual left temporal lobe than the dSPM volumes in Fig. 5C.

Given the limited differences in location and size between the dSPM 3-layer (3L) and 4-layer (4L) finite element method (FEM) epileptogenic zone (EZ) estimates, we investigated whether these small deviations were solely due to the alterations in the 4L-FEM mesh geometry or the addition of the cerebrospinal fluid (CSF) conductivity. To disentangle the geometry/conductivity effects, we set the conductivity value of the surgical cavity of the 4L-FEM mesh to match that of the brain/grey matter while all other tissue conductivity values remained the same, resulting in a mesh with a geometry of the 4L but conductivities consistent with the 3L approach. A FEM head model was computed using this “grey-matter cavity” (GM cavity) mesh with *DUNEuro* in *Brainstorm* (default FEM solver and source model settings used; volumetric head model option selected with regular isotropic cortical grid with 5mm resolution as detailed in section 2.3 Magnetoencephalography Data Acquisition and Processing)^1-4^. The resulting GM cavity FEM head model was used to derive dSPM source imaging for each of the 6 epileptiform activities included in this study. The steps used to generate this source imaging do not deviate from those described in Section 2.4 Magnetic Source Imaging concerning dSPM source estimation. A prespecified 75% of the respective maximum value threshold was applied to each dSPM MSI. Derivation of these GM cavity volumes was performed using the same practices outlined in Section 2.5.1 dSPM Source Maps. The resulting GM cavity sources were compared to the original 3L- and 4L-EZs using DICE similarity coefficients (Figure S2) to isolate and investigate potential geometry and conductivity contributions, respectively.

The DICE similarity coefficients between the 3L-FEM and GM cavity FEM sources range from 0.42 to 0.80, while those between the 4L-FEM and GM cavity FEM sources range from 0.32 to 0.91 (Figure S2). If only the geometrical mesh changes were causing the small differences, then the DICE values between the 4L-FEM and GM cavity FEM should be equal to one. Similarly, if the CSF conductivity was solely responsible, then the DICE values between the 3L-FEM and GM cavity FEM should also equal one. However, this was not true for either case, suggesting that both factors may contribute, but this auxiliary comparison does not estimate the separate effect sizes of the geometry and conductivity and therefore cannot exclude an interaction between them.

**Supplemental Figure 2:** Boxplot of the DICE similarity coefficients between the original magnetic dSPM sources derived from 3-layer FEM and 4-layer FEM head models and those derived from the 4-layer FEM mesh with the surgical cavity conductivity set to that of grey matter (GM cavity).

|  | *Pairwise dSPM Source – Centroid Distances (cm)* | | | | | |
| --- | --- | --- | --- | --- | --- | --- |
| ***Sources*** | ***A*** | ***B*** | ***Ci*** | ***Cii*** | ***Di*** | ***Dii*** |
| ***OS-BEM*** | 1.28 | 1.52 | 1.44 | 1.00 | 1.42 | 1.84 |
| ***OS-3L*** | 0.86 | 0.96 | 0.42 | 0.54 | 0.53 | 1.50 |
| ***OS-4L*** | 0.95 | 0.68 | 0.68 | 0.51 | 0.54 | 1.43 |
| ***BEM-3L*** | 0.42 | 0.63 | 1.04 | 0.58 | 1.05 | 0.80 |
| ***BEM-4L*** | 0.45 | 0.95 | 0.94 | 0.57 | 1.11 | 0.65 |
| ***3L-4L*** | 0.36 | 0.54 | 0.31 | 0.05 | 0.07 | 0.71 |

**Supplemental Table 1:** Summary table of the pairwise distances between dSPM source centroids derived from overlapping spheres (OS), boundary element method (BEM), 3-layer (3L) finite element method, and 4-layer (4L) finite element method head models. Ci = Participant C’s interictal activity, Cii = Participant C’s ictal activity, Di = Participant D’s activity from their first MEG recording, and Dii = Participant D’s activity from the second MEG recording.

|  | *Pairwise Dipole Source Distances (cm)* | | | |
| --- | --- | --- | --- | --- |
| *Sources* | ***A*** | ***Ci*** | ***Di*** | ***Dii*** |
| *OS-BEM* | 0.5 | 0.26 | 0.28 | 2.87 |
| *OS-3L* | 1.22 | 0.23 | 0.33 | 2.83 |
| *OS-4L* | 1.00 | 0.52 | 0.64 | 4.39 |
| *BEM-3L* | 0.87 | 0.07 | 0.14 | 0.50 |
| *BEM-4L* | 0.50 | 0.52 | 0.62 | 1.87 |
| *3L-4L* | 0.71 | 0.56 | 0.76 | 2.29 |

**Supplemental Table 2:** Summary table of the pairwise distances between dipole sources derived from overlapping spheres (OS), boundary element method (BEM), 3-layer (3L) finite element method, and 4-layer (4L) finite element method head models. Ci = Participant C’s interictal activity, Di = Participant D’s activity from their first MEG recording, and Dii = Participant D’s activity from the second MEG recording. No distances are provided for participant B and Cii because neither ictal dataset yielded dipoles meeting the goodness-of-fit.

|  | *Retained Dipoles per Activity and Head Model* | | | |
| --- | --- | --- | --- | --- |
| ***Head Model*** | ***A*** | ***Ci*** | ***Di*** | ***Dii*** |
| ***OS*** | 1 | 6 | 6 | 1 |
| ***BEM*** | 1 | 7 | 6 | 1 |
| ***3L*** | 1 | 7 | 6 | 1 |
| ***4L*** | 1 | 7 | 6 | 1 |

**Supplemental Table 3:** The number of dipoles derived from each head model per patient activity that survived the 70% goodness-of-fit threshold. MEG data from A and Dii were a single continuous recording, hence why there is only one dipole per head model. The MEG data for Ci and Di were split into multiple continuous segments (e.g., due to head motion), causing multiple dipoles per head model as described in Section 2.5.2 ECD Source Maps.

**Supplemental Material References**

1.	Medani T, Garcia-Prieto J, Tadel F, Antonakakis M, Erdbrügger T, Höltershinken M, et al. Brainstorm-DUNEuro: An integrated and user-friendly Finite Element Method for modeling electromagnetic brain activity. NeuroImage. 2023;267:119851.

2.	Tadel F, Baillet S, Mosher JC, Pantazis D, Leahy RM. Brainstorm: a user-friendly application for MEG/EEG analysis. Comput Intell Neurosci. 2011;2011:879716-.

3.	Medani T, Garcia-Prieto J, Tadel F, Schrader S, Antonakakis M, Joshi A, et al. Realistic head modeling of electromagnetic brain activity: an integrated Brainstorm-DUNEuro pipeline from MRI data to the FEM solutions: SPIE; 2021.

4.	Schrader S, Westhoff A, Piastra MC, Miinalainen T, Pursiainen S, Vorwerk J, et al. DUNEuro—A software toolbox for forward modeling in bioelectromagnetism. PLOS ONE. 2021;16(6):e0252431.
